# Natural Killer Cells in Antibody Independent and Antibody Dependent HIV Control

**DOI:** 10.3389/fimmu.2022.879124

**Published:** 2022-05-20

**Authors:** Nicole F. Bernard, Sanket Kant, Zahra Kiani, Cécile Tremblay, Franck P. Dupuy

**Affiliations:** ^1^Research Institute of the McGill University Health Centre, Montreal, QC, Canada; ^2^Division of Experimental Medicine, McGill University, Montreal, QC, Canada; ^3^Infectious Diseases, Immunology and Global Health Program, Research Institute of the McGill University Health Centre, Montreal, QC, Canada; ^4^Division of Clinical Immunology, McGill University Health Centre, Montreal, QC, Canada; ^5^ Centre de Recherche du Centre Hospitalier de l’Université de Montréal (CRCHUM), Montreal, QC, Canada; ^6^Department of Microbiology Infectiology and Immunology, University of Montreal, Montreal, QC, Canada

**Keywords:** HIV, elite controllers, NK cells, killer immunoglobulin-like receptors, HLA, ADCC, antibody dependent NK cell activation

## Abstract

Infection with the human immunodeficiency virus (HIV), when left untreated, typically leads to disease progression towards acquired immunodeficiency syndrome. Some people living with HIV (PLWH) control their virus to levels below the limit of detection of standard viral load assays, without treatment. As such, they represent examples of a functional HIV cure. These individuals, called Elite Controllers (ECs), are rare, making up <1% of PLWH. Genome wide association studies mapped genes in the major histocompatibility complex (MHC) class I region as important in HIV control. ECs have potent virus specific CD8^+^ T cell responses often restricted by protective MHC class I antigens. Natural Killer (NK) cells are innate immune cells whose activation state depends on the integration of activating and inhibitory signals arising from cell surface receptors interacting with their ligands on neighboring cells. Inhibitory NK cell receptors also use a subset of MHC class I antigens as ligands. This interaction educates NK cells, priming them to respond to HIV infected cell with reduced MHC class I antigen expression levels. NK cells can also be activated through the crosslinking of the activating NK cell receptor, CD16, which binds the fragment crystallizable portion of immunoglobulin G. This mode of activation confers NK cells with specificity to HIV infected cells when the antigen binding portion of CD16 bound immunoglobulin G recognizes HIV Envelope on infected cells. Here, we review the role of NK cells in antibody independent and antibody dependent HIV control.

## Introduction

Natural Killer (NK) cells are innate immune cells with anti-viral and anti-tumor activity (1). NK cells detect and respond to neighboring cells that lack “self” human leukocyte antigens (HLA). This response pattern arises from a process called education that involves the interaction of inhibitory NK cell receptors (NKR) with their self HLA ligands (2–4). Educated NK cells respond to virus-infected, stressed and damaged cells that appear to NK cells as “missing self” due to their having a reduced expression of cell surface HLA. Educated NK cells are tolerant to self-cells expressing sufficient HLA to bind inhibitory NKRs that signal NK cells to remain in a resting state. NK cell activation not only requires reduced signaling through inhibitory NKRs but also activating signals through the interaction of activating NKR with their ligands (5). NK cells can be activated directly when the integration of signals from inhibitory and activating NKRs upon binding their ligands on neighboring cells favors activation. They can also be activated in an antibody dependent (AD) manner. Modulation of NK cell activity by antibodies and their ability to interact with dendritic cells to shape adaptive immune responses positions NK cells at the intersection of innate and adaptive immunity (6). NK cells have been implicated in host responses to several viral infections, including to the human immunodeficiency virus (HIV), human cytomegalovirus, hepatitis B virus, hepatitis C virus and influenza virus (7–14). The importance of NK cells in the control of HIV infection is illustrated by the emergence of HIV mutations able to escape NK cell mediated immune pressure (15).

Treatment naïve people living with HIV (PLWH) display a wide range of HIV disease progression rates. Elite Controllers (ECs) are a subset of PLWH at one extreme of this range. They are rare, making up between 0.2% - 0.5% of PLWH. They suppress HIV viral load to below the limit of detection of standard viral load assays [<50 copies/milliliter (ml) of plasma] often maintaining high CD4^+^ T-cell counts without treatment (16–21). Viral Controllers (VCs) maintain viral loads of between 50 and either 2000 or 3000 copies/ml of plasma without treatment depending on the cohort studied (16, 18, 19, 22). ECs and VCs are subsets of a larger group known as long term non progressors (LTNPs) that make up approximately 5% of PLWH with the slowest rate of HIV disease progression. Since the term LTNPs was coined before the availability of HIV viral load assays, LTNPs are defined by their ability to maintain elevated CD4 counts for 7 or more years without treatment (16, 22).

ECs and VCs are heterogeneous groups whose classification is based on length of follow up, viral load cut off, presence/absence and frequency of viral load blips and CD4 counts. The specific criteria used to define ECs and VCs differ from one cohort to another (16, 23–25). In this review, we will use the terms “EC” to refer to PLWH who have a viral load of <50 copies/ml plasma and “VC” to refer to PLWH with a viral load of between 50 and 3000 copies/ml plasma with rare viral load blips (16). Terms such as slow progressors, LTNPs, HIV controllers (HIC) and controllers have been used to describe individuals who are not strictly ECs or VCs, as defined by these criteria. In this review, we will use the term “controller” to refer to individuals who are not ECs or VCs. The term “non-controllers” will be used to identify individuals not meeting the criteria for classification as “ECs”, “VCs” or “controllers”. Non-controllers are PLWH who are in the chronic phase of HIV infection and are either untreated, viremic progressors or treated PLWH. There also exist a group of PLWH who started anti-retroviral therapy (ART) early in infection, remained on ART for various durations and who were able to control their viral load after ART was stopped (26). As little information is available on a role for NK cells in these post-treatment controllers, they will not be discussed further in this review.

Several mechanisms have been proposed to underlie HIV control. Among these are infection with defective virus (18, 27, 28) and anti-virus-specific immune responses (29–31). HIV-specific CD8^+^ T cell responses in controllers are more polyfunctional and more likely to degranulate and release perforin and granzyme B upon stimulation than CD8^+^ T cells from non-controllers (32–34). HIV-specific CD4^+^ T cells from controllers are more likely to secrete Interleukin (IL)-2 and proliferate upon stimulation than those from non-controllers (35–37). The HIV proviral landscape in ECs differs from that in successfully treated PLWH. ECs have fewer proviral sequences than PLWH on ART, but the proportion of proviruses that are intact and likely to be replication competent is higher in EC than in treated PLWH (38). In ECs, compared to treated PLWH, HIV provirus is more likely to integrate into genetic regions characterized by deep latency, where reactivation is unlikely (38). Should reactivation occur, HIV from ECs express intact gene products targeted by the potent T cell responses often seen in this population. Some ECs can suppress HIV very early after infection, possibly before the emergence of adaptive immune responses (39, 40). Such early control suggests that innate responses may be involved in early HIV control in some ECs (41). This review will explore the role of NK cell mediated anti-HIV responses in HIV viral control.

## NKR-Ligand Pairs - Genetics, Structure and NK Cell Education

Genome wide association studies (GWAS) found several single nucleotide polymorphisms (SNPs) associated with viral load control that mapped to the major histocompatibility complex (MHC) on human chromosome 6 (42, 43). A SNP within an endogenous retroviral element associated with the gene encoding the protective HLA-B*57:01 antigen and a second one 35 kilobases upstream of *HLA-C* accounted for nearly 15% of the variation in viral load setpoint in untreated PLWH (42). A second GWAS confirmed these results and identified additional MHC class I alleles associated with protection and risk of HIV disease progression (43). These specific associations with HIV control mapped to amino acids within the MHC class I’s peptide binding groove implicating an important role for CD8^+^ T cell responses in HIV control (32–34, 43). It should be noted, however, that many HLA allotypes also interact with inhibitory killer immunoglobulin-like receptors (KIRs) expressed on NK cells and other lymphocyte subsets (44, 45).

**Figure 1:**
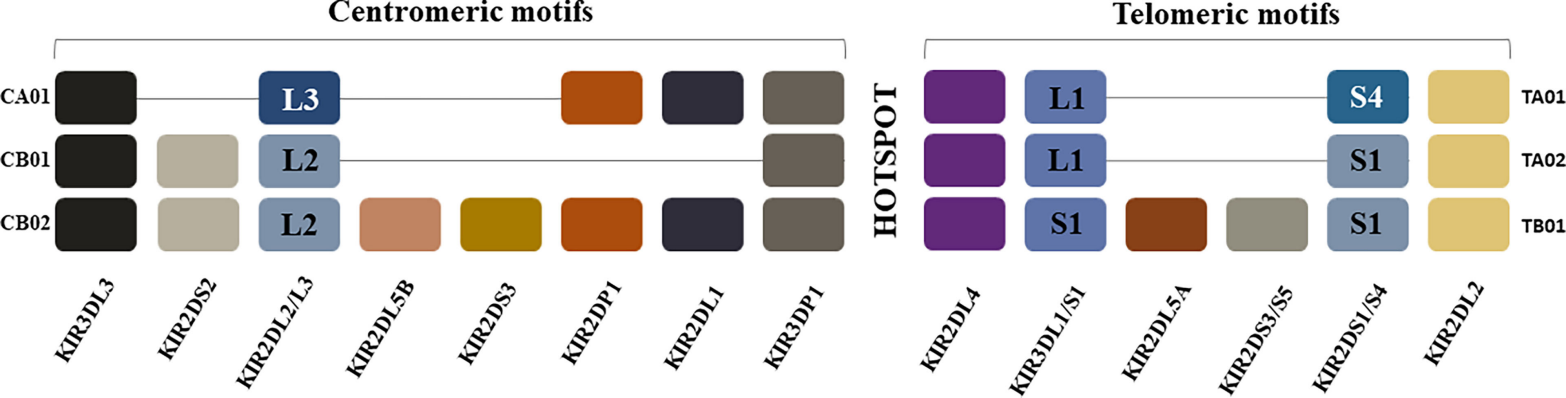
Killer Immunoglobulin-like Receptor Haplotypes. *KIR* genes are organized into haplotypes A and B. KIR haplotype A is comprised of four framework genes present in most *KIR* haplotypes (*KIR3DL3* at the centromeric end, *KIR3DL2* at the telomeric end and the pseudogene *KIR3DP1* and *KIR2DL4* in the middle) plus genes encoding inhibitory KIRs *KIR2DL1*, *KIR2DL3*, *KIR3DL1*, activating KIR *KIR2DS4* and pseudogene *KIR2DP1*. The more diverse group B haplotypes include the framework genes with various combinations of genes encoding inhibitory KIRs *KIR2DL2*, *KIR2DL5A/B* and activating KIRs *KIR2DS1*, *KIR2DS2*, *KIR2DS3*, *KIR2DS5* and *KIR3DS1*. Most *KIR* region haplotypes are composed of one of 3 centromeric and one of 3 telomeric KIR motifs that include combinations of KIR genes in linkage disequilibrium with each other, which are usually inherited together. The hotspot in the center between the centromeric and telomeric regions allows for frequent recombination between the two regions. The centromeric region is delimited by the framework genes *KIR3DL3* and *KIR3DP1* while the telomeric region is delimited by framework genes *KIR2DL4* and *KIR3DL2. KIR2DP1* and *KIR2DP1* are pseudogenes. CA01 = genes in the centromeric region of KIR haplotype A 1; CB01 = genes in the centromeric region of KIR haplotype B 1; CB02 = genes in the centromeric region of KIR haplotype B 2; TA01 = genes in the telomeric region of KIR haplotype A 1; TA02 = genes in the telomeric region of KIR haplotype A 2; TB01 genes in the telomeric region of KIR haplotype B 1.

KIR and HLA are both encoded by highly polymorphic and polygenic genetic regions on chromosomes that segregate independently from each other allowing for a great diversity of receptor ligand combinations (46, 47). KIRs are encoded by up to 15 distinct polymorphic genes within the leukocyte receptor complex on human chromosome 19q13.4 (48) (**Figure 1**). KIRs share structural features, They can have two or three extracellular immunoglobulin-like domains (i.e. 2D or 3D in the protein’s name) and either long (L) or short (S) cytoplasmic domains, transmitting inhibitory or activating signals, respectively (49). ”P” in the KIR nomenclature refers to pseudogene. Both inhibitory and activating receptors can contribute to NK cell education. Education through inhibitory receptors reduce the threshold for NK cell responses to missing-self cells while education through activating receptors dampen NK cell activation levels (2, 50).

The *KIR* gene locus is classified into *KIR-A* and *KIR-B* haplotypes, which are distinguished by their activating *KIR* gene content (**Figure 1**) (46, 51, 52). *KIR* haplotypes share 3 framework genes and 1 pseudogene. KIR3DL3 and KIR3DP1 flank the centromeric *KIR* region genes, while KIR3DL2 and KIR2DL4 flank the telomeric KIR region genes. KIR2DP1 and KIR2DL4 are separated from each other by an intergenic region, which is a recombination hotspot. The genes in the *KIR-A* haplotype encode inhibitory receptors, with the exception of *KIR2DS4* receptor, which encodes an activating receptor that is often truncated and not cell surface expressed (53, 54). *KIR-B* haplotypes include the framework genes and varying numbers of activating *KIR* genes. The genes in the telomeric and centromeric regions are in linkage disequilibrium with each other and tend to be inherited together.

The ligands for inhibitory NKR are a subset of HLA antigens (**Table 1**). KIR3DL1 is an inhibitory receptor that interacts with the subset of HLA-A and -B antigens carrying the serological Bw4 motif (55, 56). Bw4 antigens have either an isoleucine (*80I) or a threonine (*80T) at position 80 of the HLA heavy chain, which influences their cell surface expression density and ligand binding affinity (57–59). Bw4*80I variants are usually expressed at a higher density than Bw4*80T antigens (59). The remaining HLA-B antigens have a HLA-Bw6 motif that does not interact with KIR3DL1 such that NK cells expressing KIR3DL1 as their only NKR remain uneducated in *HLA*-*Bw6* homozygotes (60). The *KIR3DL1/S1* locus encodes inhibitory KIRs expressed at high (KIR3DL1-high) and low (KIR3DL1-low) cell surface densities and having no cell surface expression (KIR3DL1*004). KIR3DS1, also encoded at this locus is an activating receptor (59, 61). In general, KIR3DL1-high receptors have a higher affinity for Bw4*80I than Bw4*80T allotypes while the affinity of KIR3DL1-low receptors for Bw4*80I and Bw4*80T antigens is similar (59).

**Table 1:** NK Receptor-Ligand Pairs.

Inhibitory receptor-ligand pairs		Activating receptor-ligand pairs	
Inhibitory Receptor	Ligand	Activating receptor	Ligand
KIR2DL1	HLA-C2 group (Lys^80^)	KIR2DS1	HLA-C2 group (Lys^80^)
KIR2DL2/3	HAL-C1 (Asn^80^)	KIR2DS2	HLA-C1 group, with viral peptide
	HLA-C2 group (Lys^80^) (variable)		
KIR2DL5A/B	Unknown	KIR2DS3	unknown
KIR2DL3	Unknown	KIR2DS5	HLA-C2 (variable)
KIR3DL1	HLA-A and HLA-B allotypes with Bw4 motifs	KIR3DS1	HLA-F
KIR3DL2	HLA-A*03 and HLA-A*11	KIR2DS4	Subsets of HLA-A*03 and HLA-A*11
NKG2A/CD94	HLA class I signal peptides presented by HLA-E	KIR2DL4	HLA-G
LILRB1	HLA class I	CD16	Immunoglobulin G Fc region
		NKp30	B7 family member B7-H6, HLA-B associated transcript 3 (Bat-3)
		NKp44	heparan sulfate, proteoglycan, proliferating cell nuclear antigen
		NKp46	heparan sulfate, proteoglycan, heparin, vimentin
		NKG2C	HLA-E complexed with the peptides from the leader sequence of HLA-A, -B, -C, and -G and Human cytomegalovirus UL40

The inhibitory KIRs, KIR2DL1, KIR2DL2 and KIR2DL3 use HLA-C allotypes as ligands. HLA-C allotypes can be dichotomized into HLA-C1 and HLA-C2 groups based on whether they have an asparagine or lysine at position 80 of their heavy chain. C1 allotypes are ligands for KIR2DL3 (62). C2 allotypes are ligands for KIR2DL1 and KIR2DS1 (63–65). KIR2DL2 is an intermediate receptor, which also binds HLA-C1 and some HLA-C2 allotypes (64).

Besides KIRs, other inhibitory NKR contribute to NK cell education and tolerance to self. The NK group 2 member A (NKG2A)/CD94 heterodimer are C-type lectin receptors that recognize epitopes from the leader region of HLA-A, -B, -C and -G presented by the MHC class Ib HLA-E antigen (**Table 1**) (66–68). The leukocyte immunoglobulin-like receptor 1 (LIR-1) also interacts with conserved regions of HLA antigens (69). The signaling lymphocytic activation molecule (SLAM) family can provide NK cells with either activating or inhibitory signals (70).

**Figure 2:**
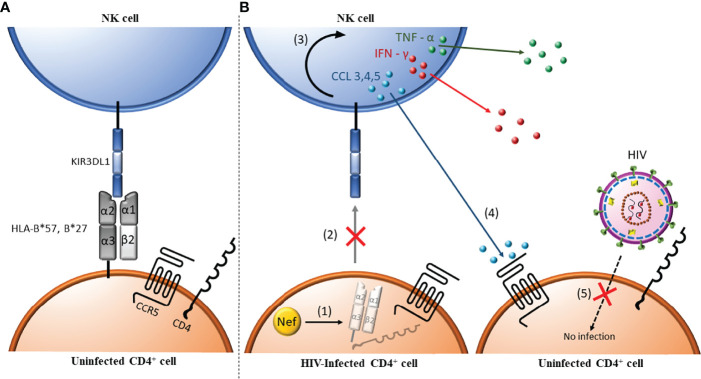
Antibody independent NK cell anti-HIV activity. (1) HIV-uninfected CD4^+^ cells express major histocompatibility complex (MHC) class I antigens (i.e. HLA-B*57/*27). These MHC class I ligands can interact with the inhibitory Killer Immunoglobulin-like Receptor (KIR) KIR3DL1. **(A)** When this interaction occurs during NK cell ontogeny, NK cells are educated to be tolerant to normal self-cells. **(B)** In HIV infected CD4 cells, Nef (orange circle) downregulates MHC I (i.e. HLA-B*57/*27), which are ligands for the inhibitory KIR3DL1 (1). Absence of the cell surface ligands for KIR3DL1 abrogates negative signaling through this receptor changing the balance between negative and positive signals received towards NK cell activation (2) and release of inflammatory cytokines such as tumor necrosis factor α (TNF-α) and interferon γ (IFN-γ) and chemokines such as CCL3, CCL4 and CCL5 (3). CCL4 (and CCL3 and CCL5) bind to CCR5, the co-receptor for HIV entry (4), which blocks the infection of uninfected, bystander CD4^+^ cells (5).

Binding of inhibitory NKRs to their HLA ligands transduces intracellular inhibitory signals that generally dominate activating ones to support tolerance to healthy HLA expressing autologous cells (5, 71, 72). NK cell education occurs when inhibitory NKR^+^ NK cells interact with healthy self HLA^+^ cells during development. Education prevents auto-reactivity to self-cells and primes NK cells for activation when they encounter target cells with reduced levels of HLA ligands as can occur in virus infected, transformed and stressed or damaged cells (73) (**Figure 2**). NK cell education levels depend on the number of inhibitory NKR-ligand interactions, inhibitory NKR-ligand density and the avidity of this interaction, which, in turn, correlates with the level of activation mature NK cells achieve when they encounter cells with aberrant HLA expression levels (2, 59, 74). NKRs are expressed stochastically on overlapping NK cell subsets. Thus, both educated and uneducated NK cells can co-exist as can NK cells with varied levels of education and responses to stimulation, due to allelic *NKR* and *HLA* variation (57, 59, 64). The inhibitory NKR^+^ NK cell subsets unable to interact with self HLA during development remain uneducated and are hyporesponsive unless activating signals predominate (4, 60, 75, 76).

## KIR/HLA Receptor Ligand Combinations in HIV Control

Epidemiological studies have identified specific *KIR/HLA* combined genotypes that influence the outcome of HIV infection (12, 13). Inhibitory KIR3DL1 and its activating KIR3DS1 counterpart are associated with slower time to the acquired immunodeficiency syndrome (AIDS) (using the 1987 definition for AIDS of the US Centers for Disease Control and Prevention), time to CD4 counts of >200 cells/mm^3^, HIV viral load control and in the case of KIR3DS1 protection against opportunistic infection late in disease, when co-expressed with certain Bw4 allotypes (12, 13, 77). In these studies, Bw6 homozygotes with uneducated KIR3DL1^+^ NK cells served as controls for the effect of educated KIR3DL1^+^ NK cells on HIV outcomes.

The protective HLA-B*57 and HLA-B*27 alleles are present in a higher frequency of controllers than non-controllers (43, 78–81). The KIR3DL1*h/*y + B*57 genotype encodes at least 1 copy of a KIR3DL1-high receptor. This combination conferred the strongest protection against HIV disease progression compared to Bw6 homozygotes (13). This is consistent with HLA-B*57’s protective effect on HIV disease outcome not only being due to its function in restricting CD8^+^ T cells responses but also to its role in mediating protective NK cell responses to HIV. While B*57 is enriched in ECs, most B*57^+^ PLWH fail to control their infection and progress to AIDS at a rate indistinguishable from that seen in PLWH without B*57 (16, 35, 78). A nonsynonymous variant of the KIR3DL1 (I47V) with a valine rather than an isoleucine at codon 47 modifies the protection conferred by B*57:01 but not B*57:03 (82). The rs643347G SNP which encodes the KIR3DL1 47V variant is associated with EC status, lower viral loads and higher CD4 counts (82).

The KIR3DL1*l/*x + B*27 combined genotype, where *l/*x encodes either two copies of KIR3DL1-low receptors or one KIR3DL1-low and a KIR3DL1*004 null receptor, also confers protection against HIV disease progression, though the protective effect is not as strong as the one associated with the KIR3DL1*h/*y + B*57 genotype combination (13). Other *KIR/HLA* combinations associated with slower time to AIDS and lower viral load than seen in *Bw6* homozygotes include KIR3DL1*004 + Bw4, KIR3DL1*h/*y + Bw4*80I, KIR3DL1*h/*y + B*27, KIR3DL1*l/*x + B*57 and KIR3DS1 + Bw4*80I (12, 13).

The involvement of HLA-C in HIV control has been controversial (16, 83). Some have questioned whether any influence attributed to HLA-C on HIV control could be explained by its linkage disequilibrium with protective HLA-B allotypes such as B*57:01 (80). On the other hand, there is support for the independent involvement of HLA-C molecules in HIV-1 infection outcomes (84, 85). GWAS studies found that SNP rs9264942 located 35 kilobases upstream of the HLA-C locus was associated with HIV viral load control (42, 43), slower progression to AIDS (86) and HLA-C cell surface expression levels in Caucasians (87). The rs9264942 SNP is marking another SNP, rs67384697 in the 3’ untranslated region of *HLA-C*, encoding either guanine or a deleted base pair at codon 263 mapping to the 3′ untranslated region of HLA-C that affects the binding of the microRNA miR-148a to this site (88). Weakly expressed HLA-C allotypes possess an intact miR-148a binding site (i.e. a guanine nucleotide at codon 263) while HLA-C antigens expressed at high levels have a deletion at this site, which prevents binding of this microRNA (88). Independent evidence for a causal effect of HLA-C expression levels on HIV control comes from the correlation between the frequency of escape mutations in predicted HLA-C epitopes and viral load (89, 90) and from the strong positive correlation between the frequency of HLA-C peptide-restricted CTL responses and HLA-C expression levels (90). It is not clear whether the immune mechanisms of HIV protection associated with these SNPs involve NK cells.

Malnati et al. compared Caucasian ECs with undetectable viral loads and LTNPs with persistent detectable viremia for genetic markers on chromosome 6 in combination with their KIR genotypes (91). They found that genotypes encoding HLA-C2 groups antigens expressed at high cell surface levels, due to their having *HLA-C2* alleles insensitive to miR-148 regulation, co-carried with telomeric *KIR-B* haplotype genes encoding activating KIR3DL1 and KIR2DS receptors were a hallmark of EC status. In contrast, homozygosity for HLA-Bw4*80I but not homozygosity for the HLA-C linked rs67384697 *243/del* SNP predicted LTNPs status. Classification as an LTNP was not associated with the presence of telomeric activating KIRs. These findings implicate a role for activating KIR2DS receptors in HIV elite control.

The ligand for the inhibitory NKG2A NKR is HLA-E, stabilized by signal peptides originating from the leader sequence of HLA-A, -B and -C molecules corresponding to amino acids -22 to -14 (66, 67, 92) (**Table 1**). Position 2 of this signal peptide can be either a methionine (-21M) or a threonine (-21T) (93, 94). All HLA-A and HLA-C isotypes are -21M, while HLA-B antigens can be dichotomized into -21M or -21T variants (94). The HLA-B -21 M/T variant distinguishes two types of HLA haplotypes, each having distinct influences on NK cell education. HLA-B -21M variants contribute more effectively to NKG2A mediated education while HLA-B -21T variants preferentially contribute to education through inhibitory KIRs (94). As *HLA-B -21M* and *HLA-Bw6/HLA-C1* are in linkage disequilibrium, the HLA antigens encoded by *-21M HLA-B* haplotypes are usually HLA-Bw6 positive, which does not interact with KIR3DL1 and HLA-C1 antigens are often expressed at low cell surface densities and interact with KIR2DL2/3 with lower avidities than do HLA-C2 with KIR2DL1, consequently affecting the potency of NK cell education and NK cell activation upon missing-self recognition.

HLA-A expression levels vary from one allotype to another (95). A higher density of HLA-A expression is associated with worse prognosis in PLWH (95). HLA-A expression levels distinguish ECs from non-controllers (95). *HLA* haplotypes that carry *HLA-B -21M* and high expression *HLA-A* alleles encode HLA antigens that preferentially provide epitopes that stabilize cell surface HLA-E expression, augmenting its cell surface expression, such as on HIV infected cells; this biases NK cell education through inhibitory NKG2A (94). Once NKG2A^+^ NK cells, educated in this manner, encounter HIV infected cells expressing high levels of HLA-E, inhibition of NK cell effector function ensues, which negatively impacts HIV control (13, 95).

In adult HIV infection, the most important immunogenetic factors contributing to time to AIDS and HIV viral load are the protective and disease-susceptible HLA-B allotypes (37, 42, 96, 97). Several *KIR3DL1 + Bw4* combinations also contribute to more favorable HIV outcomes in adults (13, 98). In contrast, in untreated pediatric HIV infection, the HLA-B antigens that are protective in adults do not significantly contribute to HIV outcomes (98). A study performed on >300 children from sub-Saharan Africa classified children as progressors, slow progressors who were viral non-controllers and slow progressors who were viral controllers. This classification was based on how old the children were when they reached pre-determined CD4 count criteria to start ART if this occurred after five years of age and whether their plasma viral load was <10,000 versus >10,000 RNA copies/ml plasma (98). Carriage of *Bw4* and low expression *HLA-A* alleles were strongly associated with HIV control and slow HIV disease progression in this pediatric cohort (98, 99). As carriage of *KIR3DS1* is rare in many African populations, Bw4 is likely co-expressed with KIR3DL1, which educates these NK cells. The viral controller children, compared to the other pediatric groups, were likely to be controlling HIV through NK cell responses using a KIR based education program.

## Links Between *KIR/HLA* Genotypes and NK Cell Functional Potential

When HLA-null cells such as 721.221 and K562 cells stimulate NK cells, negative signaling through inhibitory KIRs is abrogated while signals through activating receptors binding their ligands on HLA-null cells persist. In this scenario, NK cells are activated in accordance with the potency with which they were educated, revealing their functional potential. HLA-null cell stimulation of NK cells from carriers of the KIR3DL1-high + HLA-B*57 genotype combination activates a higher frequency of NK cells to secrete IFN-γ, the chemokine CC motif ligand 4 (CCL4) and externalize the degranulation marker CD107a than NK cells from carriers of other *KIR3DL1/HLA* combinations or from *Bw6* homozygotes (100). This is the case for NK cells from both HIV uninfected persons (100) and from PLWH (101). It should be noted, however, that HIV infection has a negative impact on NK cell functionality, though NK cells from controllers have higher functional potential than those from non-controllers (102–106).

NK cells from individuals carrying the KIR3DL1 + HLA-B*27 and KIR3DL1*004 + *HLA-Bw4* genotype combinations had higher functional potential when stimulated with HLA-null cells than those from *Bw6* homozygotes (107, 108). The latter finding suggests that the KIR3DL1-null allotype KIR3DLI*004, while not expressed on the surface of NK cells, does support NK cell education (109, 110). Korner et al. showed a similar pattern of responses to HLA-null cells for KIR2DL^+^ NK cells (111). A higher frequency of NK cells educated though KIR2DL1 and KIR2DL3 than their uneducated counterparts responded HLA-null 721.221 cell stimulation by secreting tumor necrosis factor alpha (TNF-α) and externalizing CD107a (111).

## *KIR/HLA* Genotypes Influence Responses to Autologous HIV Infected CD4 T Cells

The HIV negative regulatory factor (Nef) and the viral protein U (Vpu) are HIV encoded accessory protein with several functions, including the ability to down-modulate HLA-A, -B and -C from the surface of HIV infected cells (112–116). Most HIV isolates can reduce the levels of HLA-A and -B expression while HIV isolates differ in their ability to downmodulate HLA-C (111, 112, 114, 117). The lower HLA antigen expression levels on infected CD4 cells reduces signaling through inhibitory NKRs on autologous educated NK cells, which leads to their activation (112, 114). NK cells, activated by missing-self recognition of autologous HIV infected cells, control HIV replication, at least in part, by secreting soluble CCL3, CCL4, and CCL5, which binds the C-C chemokine receptor 5 (CCR5), the co-receptor for HIV entry, thus blocking the infection of new HIV susceptible CD4 target cells (118, 119).

Inhibitory KIR^+^ NK cells educated through interactions with their HLA ligands on neighboring cells have higher responses to autologous HIV-infected CD4 cells than their uneducated counterparts. This can be observed by gating on NK cells expressing a single inhibitory KIR by flow cytometry. The frequency of single positive KIR3DL1 NK cells secreting IFN-γ, CCL4 and externalizing CD107a, all of which have anti-HIV activity is higher in educated, than uneducated, KIR3DL1^+^ NK cells responding to autologous HIV-infected CD4 cells stimulation (117). Expression levels of both the KIR3DL1 receptor and its HLA-B ligand and their binding affinity influence single positive KIR3DL1^+^ NK cell education potency and responsiveness to autologous HIV-infected CD4 cells (59, 117). For example, NK cells from KIR3DL1-high + HLA-B*57 carriers have a superior ability to inhibit HIV replication in autologous infected CD4 cells compared to carriers of other KIR/HLA combinations and *Bw6* homozygotes (118).

Carriage of the *KIR3DS1* + HLA-Bw4*80I allele combination, associated with slower time to AIDS produces NK cells with a superior ability to suppress HIV replication in autologous HIV-infected CD4 cells compared to carriers of either the receptor or ligand alone or neither (12, 77, 120). Direct evidence for an interaction between KIR3DS1 and most HLA-Bw4*80I antigens is lacking with the exception of one report showing that KIR3DS1 interacted with HLA-B*57, if certain HIV peptides were present (121–124). KIR3DS1 binds the open conformation of the non-classical MHC class Ib antigen, HLA-F (121). NK cells stimulated by the HLA-F expressing HLA-null cell line, 721.221 and autologous HIV-infected CD4 cells, stimulated a higher frequency of NK cells to produce IFN-γ, CCL4 and CD107a than control non-HLA-F expressing stimuli (121, 125, 126). Blocking this interaction with either antibodies to HLA-F or KIR3DS1-Fc chimeric proteins significantly reduced KIR3DS1^+^ NK cell activation. Further investigations are needed to understand the anti-viral activity of KIR3DS1^+^ NK cells from individuals who are HLA-B*80I^+^ given that interactions between this receptor and ligand pair have been challenging to demonstrate experimentally.

NK cell education through HLA-C ligand/inhibitory KIR2DL receptor interactions, also contribute to responsiveness to autologous HIV infected cells. As was the case for single positive KIR3DL1 NK cells, a higher frequency of educated than uneducated single positive KIR2DL1, KIR2DL2 and KIR2DL3 NK cells responded to autologous HIV-infected CD4 cells by producing IFN-γ, CCL4 and CD107a with the strongest effect seen for CCL4 secretion (117). Similar results were observed for KIR2DL1^+^ and KIR2DL3^+^ NK cells responding to co-cultured autologous HIV infected cells, using inhibition of viral replication as a read out. Educated NK KIR2DL1^+^ and KIR2DL3^+^ cells also had a superior ability, compared to their uneducated counterparts, to reduce viral load levels in these co-cultures (111).

In summary, NK cell interactions with autologous HIV-infected CD4 cells stimulate a higher frequency of educated than uneducated NK cells to elicit functions that control HIV. The frequency of NK cells secreting CCL4 appears to be particularly important in discriminating inhibitory KIR/HLA combinations with variations in education potency (117). Larger study populations will be required to determine whether individual protective HLA alleles identified in epidemiological studies and by GWAS educate NK cells for anti-HIV activity, as was observed for HLA-null cell stimulation of NK cell (13, 42, 43, 100, 101, 117).

## Association Between NK Cell Activity and the HIV Reservoir Size

The KIR/HLA combinations discussed above educate NK cells, which then respond to autologous HIV infected cells with reduced cell surface HLA levels by stimulating functions that control HIV. HIV control is assessed by measuring viral load in supernatant, the frequency of activated NK cells producing anti-HIV functions and the frequency of HIV infected cells in NK cell/HIV infected cell co-cultures. PLWH have proviral DNA integrated into long-lived HIV reservoirs, which are an obstacle to viral clearance and HIV cure (127). ECs who exhibit the features of a functional HIV cure, are distinguished from successfully treated PLWH by having smaller HIV viral reservoirs (128). The size of the HIV proviral reservoir is thought to reflect immune mechanisms that contain HIV replication. What these mechanisms are is an active area of investigation. There is some evidence that NK cell subsets play a role in shaping the size of the integrated HIV DNA reservoir.

Using mass cytometry to measure the expression of twenty-four cell surface markers on peripheral NK cells, two machine learning approaches were used to identify NK cell subpopulations that distinguished ECs from untreated, viremic, non-controllers and that correlated with the size of integrated HIV DNA reservoir. The frequency of the CD11b^+^, CD57^-^, CD161^+^, Siglec-7^+^ subset of CD56^dim^ CD16^+^ NK cells was significantly more abundant in ECs and HIV uninfected controls than in untreated, viremic non-controllers and correlated with cell associated proviral HIV DNA levels (129). These markers identify highly active, partially mature, cytotoxic NK cells with proliferative capacity and responsiveness to cytokines such as IL-12/IL-18. This NK cell sub-population had an enhanced ability to respond to IL-12/IL-18 stimulation by secreting IFN-γ and to HLA-null cell stimulation by externalization of CD107a compared to CD11b^-^, CD161^-^ or Siglec-7^-^ CD56^dim^ CD16^+^ NK cells (129). The activity of this subpopulation may be contributing to HIV control in untreated ECs.

Marras et al. investigated NK cell functional features that were inversely correlated with total and integrated HIV DNA copy number in circulating CD4 T cells (128). The higher the frequency of NK cells induced to secrete IFN-γ following IL-2 and IL-15 stimulation and the higher the induction of activating NKp30/NKp46 natural cytotoxicity receptors over baseline by IL-2, the smaller the HIV reservoir size. The fold increase over background in induced NKp30/NKp46 expression positively correlated with the cytotoxic potential of these NK cells. NK cells from controllers and treated non-controller PLWH differed in the transcriptional program of their IL-2 induced NK cells. The functional pathways induced in NK cells from controllers upon IL-2 stimulation involved IFN-γ, IL-2/IL-12 and TNF-α signaling pathways, the nuclear factor kappa-light-chain-enhancer of activated B cells (NF-κB) pathway, lysosome signaling and cytolysis. Activated NK cells from controllers, but not from treated PLWH, dramatically suppressed viral replication and reduced levels of integrated HIV DNA in NK cell/infected CD4 T cell co-cultures (128). The control of HIV DNA copies observed predominantly in ECs supports a role for *in vivo* NK cell function in this control, even when these NK cells fail to control viral load and HIV replication.

## Antibody Dependent NK Cell Activity in HIV Control

Most CD56^dim^ NK cells express the activating immunoglobulin fragment crystallizable (Fc) gamma III a receptor, FcRγIIIa or CD16, that binds the Fc portion of immunoglobulin G (IgG), particularly to IgG1 and IgG3 subtypes (130). The antibody’s antigen binding site interacts specifically with antigens, such as those on the surface of target cells while the antibody Fc region binds and cross-links CD16 on NK cells. Antibodies thus form a bridge between NK cells and target cells, which activates NK cells to secrete cytokines, chemokines and to externalize CD107a, which are measured by AD NK cell activation (ADNKA). ADCC measures the cytolysis arising from the activation of NK cells through this interaction. Although other cell types such as macrophages, resident monocytes, neutrophils and eosinophils can also mediate ADCC, NK cells are considered the most important cell type mediating this activity.

Antibody binding to CD16 can be modified by glycosylation patterns with afucosylated antibodies having the strongest affinity for CD16 (131, 132). There can be regulation of ADCC activity at the effector cell level since there are two major allelic variants of CD16, which are distinguished by a SNP that affects ADCC activity (133, 134). A valine at amino acid 158 of CD16 confers enhanced affinity for IgG1 and IgG3 compared to a phenylalanine at this position (130). Individuals expressing the V158 variant have enhanced ADCC activity (135).

### Methodological Considerations – Antibodies, Target Cells and ADCC/ADNKA Assays

**Figure 3:**
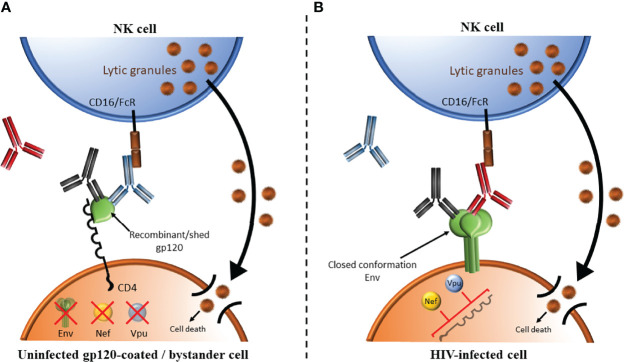
Comparison of ADCC using target cells coated with HIV Envelope gp120 versus Envelope on HIV infected cells. **(A)** HIV Envelope gp120-CD4 interactions produce an open Envelope conformation exposing CD4 induced (CD4i) epitopes that are hidden in trimeric closed conformation HIV Envelope. This occurs when target cells are gp120-coated cells or bystander CD4^+^ cells binding gp120 shed from co-cultured HIV infected cells. This conformation can be recognized by antibodies to CD4i epitopes (shown in blue), some broadly neutralizing antibodies (BnAbs, shown in black) but not by most BnAbs (shown in red). In gp120-coated cells, the HIV proteins Envelope (green), Nef (orange circle) and Vpu (light blue circle) are absent. The Fc portion of antibodies to the CD4i epitope bind the activating NK cell receptor, CD16/FcR, which activates NK cells to secrete cytokine, chemokines and lytic granules to kill target cells by ADCC. **(B)** Nef and Vpu encoded by HIV downmodulate CD4 preventing CD4 from interacting with HIV Envelope. The Envelope is thus presented on the surface of HIV infected cells is its closed conformation. BnAs shown in red and black recognize this closed Envelope conformation while antibodies to the CD4i epitope shown in blue do not. The Fc portion of Envelope bound antibodies binds CD16/FcR to activate NK cells to secrete cytokine, chemokines and lytic granules to kill target cells by ADCC.

AD functional assays targeting HIV infected cells depend on the presence of antibodies specific for HIV Envelope, the only gene product expressed on the surface of infected cells and virions (136). Anti-HIV Envelope-specific antibodies are present in all PLWH as well as in recipients of vaccines that include Envelope gp120 antigens in the vaccine regimen (137–141). Most assays measuring the ADCC competence of antibodies in PLWH use either CD4^+^ CEM.NKR.CCR5 (CEM) cells coated with monomeric recombinant HIV Envelope gp120 (142–151) or HIV-infected cells (152–155) as target cells. In both cases, CD4-induced (CD4i) epitopes that are normally hidden within the native trimeric Envelope expressed on genuinely HIV infected cells are exposed while the CD4 binding site (CD4bs) epitopes are occluded by binding CD4 expressed on coated cells CEM cells or on uninfected CD4 bystander cells present in the same culture as infected CD4 cells (156). The Envelope’s CD4bs epitopes are highly conserved and antibodies to this epitope are among the most potent broadly neutralizing antibodies (**Figure 3**). HIV Envelope gp120-CD4 interactions produce an open Envelope conformation exposing CD4i epitopes that are hidden in trimeric closed conformation HIV Envelope (140, 157). CD4i epitopes are exposed on gp120-coated target cells and bystander CD4^+^ cells that have bound gp120 shed from co-cultured HIV infected cells (156, 158). The open Envelope conformation is recognized by antibodies to CD4i epitopes such as A32, some broadly neutralizing antibodies but not by most broadly neutralizing antibodies (154–156). Since gp120-coated CEM cells are not infected, the HIV Envelope, Nef and Vpu are absent. The Fc portion of antibodies to the CD4i epitope binds the activating NK cell receptor, CD16, which activates NK cells to secrete cytokine, chemokines and externalize lytic granules to kill target cells by ADCC. In infected cells, HIV Nef and Vpu downmodulate CD4 preventing it from interacting with HIV Envelope (159). The Envelope is thus presented on the surface of HIV infected cells in its closed conformation (155, 156). Most broadly neutralizing antibodies recognize the closed Envelope conformation while antibodies to the CD4i epitope do not (155, 156). The Fc portion of Envelope bound antibodies bind CD16 to activate NK cells to secrete cytokine, chemokines and lytic granules to kill target cells by ADCC. We generated HIV-infected CEM cells that were sorted to exclude uninfected bystander cells. The advantage of using these sorted, infected CEM (siCEM) cells as target cells is that they express Envelope in a closed conformation that resembles what is present on genuinely HIV-infected cells in which the CD4i epitope is hidden and CD4bs epitopes are exposed (155) (**Figure 3**). Head-to-head comparisons of ADCC assay results using gp120-coated CEM and siCEM cells correlate with each other. However, the abundance of antibodies specific for gp120 on coated CEM cells is 7-fold or more, higher than that of antibodies specific for the closed Envelope conformation on siCEM cells (155, 160).

A plethora of assays have been developed to measure ADCC activity. The earliest assays used was ^51^Chromium release from gp120-coated target as a read out for ADCC activity. Some of the disadvantages of the ^51^Chromium release assay include the use of radioisotopes and challenges related to the quantification of target cell death. Using ^51^Chromium release assays, plasma from controllers and untreated, viremic non-controllers bound similarly to a panel of HIV Envelope antigens. Higher ADCC activity was detected in plasma from controllers that non-controllers using gp120-coated CEM cells (146). The flow cytometry-based rapid and fluorometric ADCC (RFADCC) assay has been widely used to measure ADCC activity (161). This assay measures the loss of carboxyfluorescein succinimidyl esther dye from gp120-coated CEM target cells also stained with the lipophilic membrane dye, PKH-26 (161). The RFADCC assay was eventually shown to measure monocyte mediated AD cellular trogocytosis (ADCT) rather than NK cell mediated ADCC activity as it detected the uptake of PKH-26-stained membranes from the target cells by monocyte effector cells (162). The high throughput, flow cytometry-based GranToxiLux assay measures the frequency of target cells that have taken up granzyme B released from activated NK cells (163). ADCC activity can also be evaluated by measuring the disappearance of live HIV-infected p24^+^ target cells by flow cytometry, or decreased luciferase activity when target cells are infected with luciferase-based HIV constructs, or more directly, by measuring apoptosis induction in target cells (139, 140, 154, 155, 164–167). The ADNKA assay measures NK cell activation for secretion of IFN-γ and externalization of CD107a; in some assays, secretion of TNF-α and/or CCL4 are also measured (105, 168).

## ADCC and ADNKA Activity in HIV Control

Madhavi et al. compared plasma from 22 controllers and 44 untreated, viremic non-controllers using several ADCC and ADNKA assays (147). Controllers, compared to untreated, viremic non-controllers, had higher levels of gp120-specific antibodies capable of 1) binding CD16, 2) inducing IFN-γ secretion and/or CD107a externalization from NK cells in an ADNKA assay and 3) generating granzyme B positive Envelope gp140-coated CEM cells using the GranToxiLux assay (147). The higher level of ADCC activity in plasma from controllers than in untreated, viremic non-controllers measured using the GranToxiLux assay was confirmed by others (163, 169).

In contrast, Ackerman et al. compared ECs VCs, untreated, viremic non-controllers and treated non-controllers in chronic phase infection for antibodies binding to gp120-coated beads, for activity in the RFADCC assay and for ADNKA activity using gp120-coated CEM cells opsonized with IgG isolated from study subject plasma to stimulate NK cells (142). The titer of plasma anti-gp120 IgG was lower in ECs than that in VCs and untreated, viremic non-controllers. No between-group differences were observed in the RFADCC and ADNKA assays, with the exception that ECs, compared to VCs, had lower activity in the RFADCC assay and a lower frequency of NK cells secreting CCL4 (142). To our knowledge, these functional results were not normalized to the concentration of gp120-specific antibodies present in each of the samples tested.

Using a different study population meeting the same classification criteria, Kant et al. evaluated the concentration of anti-gp120- (using gp120 coated CEM target cells) and anti-Envelope- specific antibodies (using siCEM target cells) in plasma from PLWH. ADCC activity was evaluated using an assay that measured target cell apoptosis, which correlates with target cell cytolysis (138, 155, 170). The concentration of antibodies in plasma from ECs, VCs and untreated, viremic non-controllers that bound gp120-coated CEM cells and Envelope on siCEM cells was similar and higher than the concentration of these antibodies in treated non-controllers. This was also the case for ADCC activity (155). When ADCC assay results were normalized to the concentration of anti-gp120- and anti-Envelope-specific antibodies in each plasma sample, all between-group differences fell below the level of significance. This finding suggested that between-group differences in ADCC competent antibodies were driven by differences in the concentration of the antibodies in plasma able to bind the closed conformation of HIV Envelope on siCEM cells. Thus, the four subject groups did not differ from each other in their per-anti-Envelope-specific antibody ADCC potency (138). Of particular interest, ECs and VCs with an HIV reservoir size that was below the limit of detection had significantly higher normalized ADCC activity measurements than those with a detectable HIV reservoir size, implicating a role for ADCC activity in HIV reservoir size control (138). Others have reported that production of IFN-γ by NK cells was associated with lower viral load (171–174) and lower HIV reservoir size (128).

More recent studies have expanded the AD functions tested to include AD cellular phagocytosis (ADCP), AD neutrophil phagocytosis (ADNP), AD complement deposition (ADCD), and ADCT in addition to ADNKA in one study (142) and ADCP, ADCT and ADCD in another (138). Comparisons of these activities in ECs, VCs, untreated, viremic non-controllers and treated non-controllers found minor between-group differences in ADCD and CCL4 secretion (142). As for ADCC activity, plasma from ECs, VCs and untreated, viremic non-controllers generated higher levels of ADCP, ADCT and ADCD activity than plasma from treated non-controllers (138). Between-group differences were lost upon normalizing these values to the concentration of anti-gp120-specific antibodies for ADCP, and anti-Envelope-specific antibodies for ADCT and ADCD. One clear difference in the patterns of anti-gp120- and anti-Envelope-specific antibodies and in AD functions detected in ECs, VCs, untreated, viremic non-controllers and treated non-controllers was that they were highly correlated with each other in ECs but not in the other groups (138, 142).

A systems serology approach to assess antibody features associated with HIV control included assessments of the antibody specificity to a panel of HIV protein variants, the ability of these antibodies to recruit innate immune cells by determining their titer, Ig class and subclass, antibody Fc glycosylation patterns, antibody binding to Fc gamma receptors, lectin binding properties and AD functionality (175). Anti-HIV-specific antibodies with bisected glycoforms were enriched in ECs compared to untreated, viremic progressors. Gp120-specific antibodies with these glycosylation patterns have a greater capacity to bind CD16 and mediate ADCC. Antibodies specific for HIV p24 were enriched in ECs compared with treated non-controllers as were Fc gamma receptor ligating antibodies to internal HIV gene products other than HIV capsid (175). These observations confirmed previous findings but have the potential to identify novel features of humoral immunity that differentiate ECs from non-controllers, whether on-ART or not. The interest of applying systems serology approaches to compare controllers to non-controllers is its potential to generate new information on what antibody features define functionally potent immunity and to contribute to the identification of mechanisms that correlate with potent AD effector function.

## Role of NK Cell Education in ADCC and ADNKA Activity

**Figure 4:**
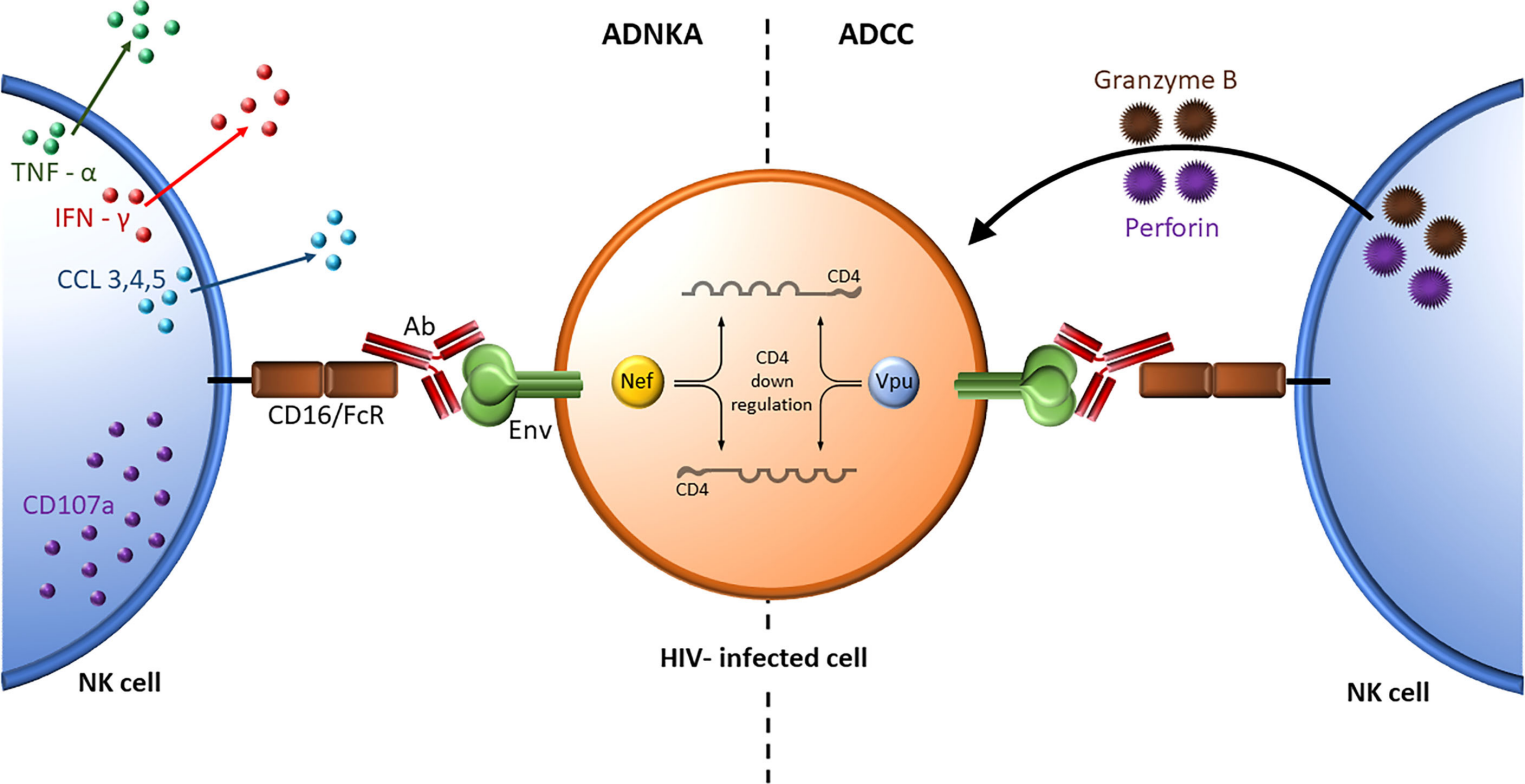
ADNKA and ADCC activity. Nef- and Vpu-mediated downregulation of cell surface CD4 prevents the interaction of CD4 with HIV Envelope leaving it in a closed trimeric conformation on the HIV infected cell surface. The antigen combining site of BnAbs (shown in red) bind this Envelope conformation while the Fc region of the BnAbs bind the activating receptor CD16/FcR on NK cells. ADNKA assesses the secretion of inflammatory cytokines such as TNF-α (green) and IFN-γ (red), and expression of the degranulation marker CD107a (purple) from NK effector cells (left) whereas ADCC assesses the cytopathic effects of cytolytic granules containing perforin and granzyme B released by NK cells on antibody opsonized target cells (right).

Although many reports refer to ADNKA and ADCC activity interchangeably, the former measures NK effector cell activation while the later measures the consequences of NK cell activation at the target cell level (**Figure 4**). The role NK cell education in ADCC and ADNKA activity was investigated. ADCC activity was measured using a GranToxiLux assay that assessed the frequency of granzyme B positive gp120-coated CEM target cells opsonized with the same source of antibodies recognizing HIV gp120. The effector cells were NK cells negatively sorted for a single inhibitory KIR that originated from subjects expressing, or not, HLA antigens able to educate NK cells that expressed the inhibitory KIR being sorted for. No differences were observed between educated and uneducated NK cells isolated for a single inhibitory KIR. This was the case for KIR3DL1, KIR2DL1 and KIR2DL2. This finding suggested that education does not play an important role in ADCC activity (176, 177). The reason for this may be due to signaling through the CD16 activating receptor overwhelming any effect on NK cell responsiveness resulting from NK cell education. ADNKA activity was measured as the frequency of NK cells positive for IFN-γ, CCL4 and/or CD107a following stimulation by opsonized gp120-coated CEM cells. Unlike ADCC activity, a consistently higher frequency of educated, than uneducated, NK cells responded to this stimulus (176). Together these results underline important differences in these two NK cell functions, which are often referred to interchangeably in the literature (178).

## Adaptive NK Cells in HIV Viral Control

Human cytomegalovirus (HCMV) infection expands a population of NK cells with adaptive-like features (8). These adaptive NK (adapNK) cells express the activating receptor, NKG2C, which belongs to the C-type lectin family (179). The NKG2C activating receptor, like its inhibitory counterpart, NKG2A, is expressed as a heterodimer with CD94 (180). The ligand for NKG2C and NKG2A is HLA-E, a nonclassical MHC class Ib antigen cell surface stabilized by peptides derived from leader sequence of classical MHC class I antigens, the nonclassical MHC Ib HLA-G antigen or epitopes derived from the HCMV encoded viral protein UL40 (**Table 1**) (66, 68, 181). The interaction of NKG2C with its ligand transmits signals that activate cells expressing this receptor (66, 182). AdapNK cells undergo DNA methylation-dependent epigenetic modifications, which distinguish them from conventional NK cells and influence their functionality (183, 184).

Some individuals do not express cell surface NKG2C due to a homozygous deletion of approximately 16 kb that includes the *nkg2c* gene encoding NKG2C (185, 186). In several Caucasian populations, a Japanese and a Tanzanian cohort, the frequency of the *NKG2C* deletion allele is close to 20% with a homozygous deletion genotype frequency of approximately 4% (186–189). Several studies have questioned whether NKG2C^+^ cells play a role in HIV viral control in PLWH.

Treatment-naive viral load set point is associated with the rate of HIV disease progression, as measured by time to CD4 counts of <200/mm^3^, AIDS, and death (190, 191). Thomas et al. found a positive correlation between HIV viral load and the proportion of NKG2C^+^ NK cells enumerated at the same time viral load was assessed in a group of 6 *NKG2C^+/+^* PLWH (192). HCMV status was not assessed in these individuals, which would influence the frequency of NKG2C^+^ NK cells. However, most PLWH are HCMV co-infected (193). Ma et al. found a negative correlation between the percentage of NKG2C^+^ NK cells and concurrent viral load in 22 treatment-naive PLWH infected for at least 120 days, which corresponded to the viral load set point (194). Gondois-Rey et al. also found a negative correlation between the percentage of NKG2C^+^ NK cells and concurrent viral load in 18 treatment-naive PLWH tested at time points in acute/early HIV infection (172). Alsulami et al. assessed the pre-treatment viral load set point in 160 NKG2C^+/+^, 83 NKG2C^+/-^, and 6 NKG2^-/-^ PLWH. They found that HIV viral load set points were similar in carriers of the three NKG2C genotypes (137). They also correlated plasma viral load in 43 HCMV seropositive PLWH with the frequency of NKG2C^+^ CD56^dim^ NK cells and the intensity of NKG2C expression on these cells. Neither the percentage of NKG2C^+^ CD56^dim^ NK cells nor the intensity of NKG2C expression on these cells was significantly correlated with viral load set point when all observations were considered together or when results were stratified according to NKG2C genotype. The later results suggest no association between the frequency of NKG2C^+^ NK cells or the intensity of NKG2C expression on NK cells with viral load control. However, as there are inconsistencies in this finding these results need to be confirmed.

## A Role for NK Cells in Vaccine Responses Able to Control HIV

The only HIV vaccine trial to show significant though modest efficacy against HIV infection was the RV144 HIV vaccine trial (195). Correlates of protection analyses revealed that vaccine induced antibodies able to bind HIV Envelope structures and support NK cell mediated ADCC activity were associated with protection from HIV infection (196). However, a similar vaccine strategy used in a South African population, which induced similar immune responses failed to show efficacy in preventing HIV infection (197). This highlights the importance of investigating strategies that can improve the efficacy of vaccine induced immune responses. ECs spontaneously control HIV without treatment. ECs, compared to non-controllers, are more likely to have polyfunctional HIV-specific T cell responses and highly correlated polyfunctional anti-Envelope antibody dependent activity (32, 142, 170). The RV144 trial provides an example of a role for NK cells in protection from HIV infection while immune responses distinguishing ECs from non-controller implicate a role for NK cells in HIV control.

Early in an immune response, NK cells secrete cytokines and chemokines such as IFN-γ, TNF-α, CCL3, CCL4 and CCL5 and granulocyte‐macrophage colony-stimulating factor (198). These secreted factors contribute to recruiting and activating antigen presenting cells such as dendritic cells, which in turn induce adaptive immunity (199). IFN-γ derived from NK cells can promote type 1 T helper cell differentiation and stimulate isotype class switching in B cells (200, 201). Designing vaccines able to harness the activity of NK cells may be able to improve the induction of adaptive immune responses able to exert HIV control (202), generate innate immune memory (203), and collaborate with other vaccine induced immune responses to increase the prophylactic and therapeutic potential of HIV vaccines (204). The incorporation of novel adjuvants into vaccine formulations may induce NK cell activities that increase adaptive immune responses (205–208).

Most PLWH are also HCMV co-infected (193). CMV infection drives the expansion of adaptive-like NK cells expressing NKG2C and/or that are negative for the FcRγ signaling molecule. These adaptive-like NK cells have epigenetic changes that confer AD effector functions such as IFN-γ secretion and ADCC activity that is superior to that seen in conventional NK cells (8, 183, 184, 209, 210). Vaccines designed to induce adaptive-like NK cells together with HIV-specific antibodies would be an interesting strategy to consider for controlling several pathogens including HIV and HCMV.

However, NK cells can also negatively regulate immune responses through their cytolytic activity. NK cell mediated cytolysis can reduce vaccine antigen persistence, eradicate responding T cells, suppress the establishment of adaptive memory and block the evolution of antibody responses (199, 211, 212). The dichotomous activity of NK cells adds complexity to targeting these cells through vaccination. Future investigations will need to consider this issue when designing vaccines targeting NK cells.

## Concluding Remarks and Future Directions

Although HIV-specific CD8 T cell responses play a predominant role in HIV control, particularly in adults, there is accumulating and compelling evidence from epidemiological, genetic and functional studies that educated NK cells also contribute to HIV control. In fact, in children, NK cells educated through inhibitory KIR play a more important role than CD8 responses restricted by protective HLA allotype. Certain NK cell subsets are also associated with EC status through less well-defined mechanisms.

A survey of the literature on AD activities in controllers (ECs, VCs) compared to untreated, viremic non-controllers and in some cases treated non-controllers has revealed few consistent patterns of AD functions associated with control other than ECs having more coordinated AD functions than other groups of PLWH. The discrepancies in whether and how AD activities differ in these groups of PLWH may be due to the assays used, what they measure and how results are analyzed. Quantification of antibodies specific for gp120 on coated CEM cells or specific for Envelope on siCEM cells and using these values to normalize AD function results should be applied. The reason for this is that the titer of antibodies specific for monomeric gp120 and trimeric Envelope vary from person to person. ECs, VCs, and untreated, viremic non-controllers have higher titers of these antibodies than do treated non-controllers. The observation that ECs and VCs with undetectable HIV DNA reservoir sizes in circulating CD4^+^ T cells have higher levels of ADCC activity than in ECs and VCs with measurable HIV reservoir sizes supports a role for ADCC in HIV control at the HIV reservoir level.

As innate immune cells, NK cells are primed to participate in anti-viral responses and exert antibody independent anti-viral activity at the earliest times post infection and certainly earlier than anti-viral T cell activity can be induced. NK cells can only exercise anti-viral functions through ADCC and ADNKA once antibodies specific for HIV gp120/Envelope appear. Future research should more thoroughly evaluate whether antibody independent NK cell responses are more likely to contribute to the functional HIV cure phenotype exhibited by ECs than AD activities. Future investigations of the anti-HIV activity of NK cells should include the evaluation of HIV reservoir size in addition to viral load in plasma and co-cultures, viral replication, and the frequency of circulating HIV infected cells. It is important at this time to remain open to the possibility that multiple NK cell dependent mechanisms may be involved in HIV control in ECs.

An interesting feature of ECs, compared to treated PLWH, is their ability to maintain high levels of potent anti-gp120- and anti-Envelope-specific antibodies with ADNKA/ADCC functionality in the absence of detectable viremia. Initiation of ART in untreated, viremic non-controllers leads to a decline in HIV-specific immune responses as seen in treated non-controllers (170). This may in part be due to the higher levels of gp-120/Envelope-specific memory B cells maintained in ECs compared to treated PLWH (213–215). This would be an important area for further investigation.

There are many challenges to these studies. Controllers, particularly ECs, are a rare subset of PLWH, impeding recruitment of large EC populations. Furthermore, is has become clear that ECs are a heterogeneous population further hampering the recruitment of homogeneous populations for investigation. There are discrepancies from one cohort to another in how controllers are defined in terms of their viral load cut offs, CD4 counts, length of follow up, among other parameters. The wide range of assays used to measure AD functions, the target cells used to measure ADCC activity and the use of ADCC and ADNKA interchangeably likely also contribute to discrepant results between studies. Overall, the assays used to assess NK cell mediated AD functionality require further standardization in order to better elucidate the role of NK cells in mediating HIV control.

## Author Contributions

NB, SK and FD performed the literature review. NB prepared the first draft of the manuscript. SK, ZK, CT, and FD were involved in critical discussions of the content, writing, and edited of the manuscript. SK, FD and NB were involved in the preparation of figures. All authors contributed to the manuscript and approved the submitted version.

## Funding

This work was supported by Canadian Institute for Health Research (CIHR) operating grant MOP-142494 and project grant # PJT-148491 and by the Fonds de Recherche du Quebec-Santé (FRQ-S) AIDS and Infectious Diseases Network grant.

## Conflict of Interest

The authors declare that the research was conducted in the absence of any commercial or financial relationships that could be construed as a potential conflict of interest.

## Publisher’s Note

All claims expressed in this article are solely those of the authors and do not necessarily represent those of their affiliated organizations, or those of the publisher, the editors and the reviewers. Any product that may be evaluated in this article, or claim that may be made by its manufacturer, is not guaranteed or endorsed by the publisher.

## References

[1] Trinchieri G. Biology of Natural Killer Cells. Adv Immunol (1989) 47:187–376. doi: 10.1016/S0065-2776(08)60664-1 2683611 PMC7131425

[2] Boudreau JE Hsu KC. Natural Killer Cell Education and the Response to Infection and Cancer Therapy: Stay Tuned. Trends Immunol (2018) 39(3):222–39. doi: 10.1016/j.it.2017.12.001 PMC6013060 29397297

[3] Brodin P Karre K Hoglund P. Nk Cell Education: Not an on-Off Switch But a Tunable Rheostat. Trends Immunol (2009) 30(4):143–9. doi: 10.1016/j.it.2009.01.006 19282243

[4] Kim S Poursine-Laurent J Truscott SM Lybarger L Song YJ Yang L. Licensing of Natural Killer Cells by Host Major Histocompatibility Complex Class I Molecules. Nature (2005) 436(7051):709–13. doi: 10.1038/nature03847 16079848

[5] Long EO Kim HS Liu D Peterson ME Rajagopalan S. Controlling Natural Killer Cell Responses: Integration of Signals for Activation and Inhibition. Annu Rev Immunol (2013) 31:227–58. doi: 10.1146/annurev-immunol-020711-075005 PMC3868343 23516982

[6] Munz C Steinman RM Fujii S. Dendritic Cell Maturation by Innate Lymphocytes: Coordinated Stimulation of Innate and Adaptive Immunity. J Exp Med (2005) 202(2):203–7. doi: 10.1084/jem.20050810 PMC2213015 16027234

[7] Oliviero B Varchetta S Paudice E Michelone G Zaramella M Mavilio D. Natural Killer Cell Functional Dichotomy in Chronic Hepatitis B and Chronic Hepatitis C Virus Infections. Gastroenterology (2009) 137(3):1151–60. doi: 10.1053/j.gastro.2009.05.047 19470388

[8] Guma M Cabrera C Erkizia I Bofill M Clotet B Ruiz L. Human Cytomegalovirus Infection Is Associated With Increased Proportions of Nk Cells That Express the Cd94/Nkg2c Receptor in Aviremic HIV-1-Positive Patients. J Infect Dis (2006) 194(1):38–41. doi: 10.1086/504719 16741880

[9] Guma M Angulo A Vilches C Gomez-Lozano N Malats N Lopez-Botet M. Imprint of Human Cytomegalovirus Infection on the Nk Cell Receptor Repertoire. Blood (2004) 104(12):3664–71. doi: 10.1182/blood-2004-05-2058 15304389

[10] Ahlenstiel G Martin MP Gao X Carrington M Rehermann B. Distinct Kir/Hla Compound Genotypes Affect the Kinetics of Human Antiviral Natural Killer Cell Responses. J Clin Invest (2008) 118(3):1017–26. doi: 10.1172/JCI32400 PMC2214845 18246204

[11] Khakoo SI Thio CL Martin MP Brooks CR Gao X Astemborski J. Hla and Nk Cell Inhibitory Receptor Genes in Resolving Hepatitis C Virus Infection. Science (2004) 305(5685):872–4. doi: 10.1126/science.1097670 15297676

[12] Martin MP Gao X Lee JH Nelson GW Detels R Goedert JJ. Epistatic Interaction Between Kir3ds1 and Hla-B Delays the Progression to Aids. Nat Genet (2002) 31(4):429–34. doi: 10.1038/ng934 12134147

[13] Martin MP Qi Y Gao X Yamada E Martin JN Pereyra F. Innate Partnership of Hla-B and Kir3dl1 Subtypes Against HIV-1. Nat Genet (2007) 39(6):733–40. doi: 10.1038/ng2035 PMC4135476 17496894

[14] Savoy SKA Boudreau JE. The Evolutionary Arms Race Between Virus and Nk Cells: Diversity Enables Population-Level Virus Control. Viruses (2019) 11(10):1–17. doi: 10.3390/v11100959 PMC6832630 31627371

[15] Alter G Heckerman D Schneidewind A Fadda L Kadie CM Carlson JM. HIV-1 Adaptation to Nk-Cell-Mediated Immune Pressure. Nature (2011) 476(7358):96–100. doi: 10.1038/nature10237 21814282 PMC3194000

[16] Deeks SG Walker BD. Human Immunodeficiency Virus Controllers: Mechanisms of Durable Virus Control in the Absence of Antiretroviral Therapy. Immunity (2007) 27(3):406–16. doi: 10.1016/j.immuni.2007.08.010 17892849

[17] Lambotte O Boufassa F Madec Y Nguyen A Goujard C Meyer L. HIV Controllers: A Homogeneous Group of HIV-1-Infected Patients With Spontaneous Control of Viral Replication. Clin Infect Dis (2005) 41(7):1053–6. doi: 10.1086/433188 16142675

[18] Gonzalo-Gil E Ikediobi U Sutton RE. Mechanisms of Virologic Control and Clinical Characteristics of HIV+ Elite/Viremic Controllers. Yale J Biol Med (2017) 90(2):245–59. PMC5482301 28656011

[19] El-Far M Kouassi P Sylla M Zhang Y Fouda A Fabre T. Proinflammatory Isoforms of Il-32 as Novel and Robust Biomarkers for Control Failure in HIV-Infected Slow Progressors. Sci Rep (2016) 6:22902. doi: 10.1038/srep22902 26978598 PMC4792165

[20] Okulicz JF Lambotte O. Epidemiology and Clinical Characteristics of Elite Controllers. Curr Opin HIV AIDS (2011) 6(3):163–8. doi: 10.1097/COH.0b013e328344f35e 21502920

[21] Blankson JN. Control of HIV-1 Replication in Elite Suppressors. Discov Med (2010) 9(46):261–6. 20350494

[22] Okulicz JF Marconi VC Landrum ML Wegner S Weintrob A Ganesan A. Clinical Outcomes of Elite Controllers, Viremic Controllers, and Long-Term Nonprogressors in the Us Department of Defense HIV Natural History Study. J Infect Dis (2009) 200(11):1714–23. doi: 10.1086/646609 19852669

[23] Olson AD Meyer L Prins M Thiebaut R Gurdasani D Guiguet M. An Evaluation of HIV Elite Controller Definitions Within a Large Seroconverter Cohort Collaboration. PloS One (2014) 9(1):e86719. doi: 10.1371/journal.pone.0086719 24489776 PMC3904947

[24] Gurdasani D Iles L Dillon DG Young EH Olson AD Naranbhai V. A Systematic Review of Definitions of Extreme Phenotypes of HIV Control and Progression. AIDS (2014) 28(2):149–62. doi: 10.1097/QAD.0000000000000049 PMC3882304 24149086

[25] Chereau F Madec Y Sabin C Obel N Ruiz-Mateos E Chrysos G. Impact of Cd4 and Cd8 Dynamics and Viral Rebounds on Loss of Virological Control in HIV Controllers. PloS One (2017) 12(4):e0173893. doi: 10.1371/journal.pone.0173893 28380038 PMC5381858

[26] Saez-Cirion A Bacchus C Hocqueloux L Avettand-Fenoel V Girault I Lecuroux C. Post-Treatment HIV-1 Controllers With a Long-Term Virological Remission After the Interruption of Early Initiated Antiretroviral Therapy Anrs Visconti Study. PloS Pathog (2013) 9(3):e1003211. doi: 10.1371/journal.ppat.1003211 23516360 PMC3597518

[27] Blankson JN Bailey JR Thayil S Yang HC Lassen K Lai J. Isolation and Characterization of Replication-Competent Human Immunodeficiency Virus Type 1 From a Subset of Elite Suppressors. J Virol (2007) 81(5):2508–18. doi: 10.1128/JVI.02165-06 PMC1865922 17151109

[28] Wang B Dyer WB Zaunders JJ Mikhail M Sullivan JS Williams L. Comprehensive Analyses of a Unique HIV-1-Infected Nonprogressor Reveal a Complex Association of Immunobiological Mechanisms in the Context of Replication-Incompetent Infection. Virology (2002) 304(2):246–64. doi: 10.1006/viro.2002.1706 12504566

[29] Pernas M Casado C Arcones C Llano A Sanchez-Merino V Mothe B. Low-Replicating Viruses and Strong Anti-Viral Immune Response Associated With Prolonged Disease Control in a Superinfected HIV-1 Ltnp Elite Controller. PloS One (2012) 7(2):e31928. doi: 10.1371/journal.pone.0031928 22384103 PMC3286446

[30] Edwards BH Bansal A Sabbaj S Bakari J Mulligan MJ Goepfert PA. Magnitude of Functional Cd8+ T-Cell Responses to the Gag Protein of Human Immunodeficiency Virus Type 1 Correlates Inversely With Viral Load in Plasma. J Virol (2002) 76(5):2298–305. doi: 10.1128/jvi.76.5.2298-2305.2002 PMC135950 11836408

[31] Berger CT Frahm N Price DA Mothe B Ghebremichael M Hartman KL. High-Functional-Avidity Cytotoxic T Lymphocyte Responses to Hla-B-Restricted Gag-Derived Epitopes Associated With Relative HIV Control. J Virol (2011) 85(18):9334–45. doi: 10.1128/JVI.00460-11 PMC3165743 21752903

[32] Betts MR Nason MC West SM De Rosa SC Migueles SA Abraham J. HIV Nonprogressors Preferentially Maintain Highly Functional HIV-Specific Cd8+ T-Cells. Blood (2006) 107(12):4781–9. doi: 10.1182/blood-2005-12-4818 PMC1895811 16467198

[33] Almeida JR Price DA Papagno L Arkoub ZA Sauce D Bornstein E. Superior Control of HIV-1 Replication by Cd8+ T Cells Is Reflected by Their Avidity, Polyfunctionality, and Clonal Turnover. J Exp Med (2007) 204(10):2473–85. doi: 10.1084/jem.20070784 PMC2118466 17893201

[34] Migueles SA Laborico AC Shupert WL Sabbaghian MS Rabin R Hallahan CW. HIV-Specific Cd8+ T Cell Proliferation Is Coupled to Perforin Expression and Is Maintained in Nonprogressors. Nat Immunol (2002) 3(11):1061–8. doi: 10.1038/ni845 12368910

[35] Emu B Sinclair E Hatano H Ferre A Shacklett B Martin JN. Hla Class I-Restricted T-Cell Responses May Contribute to the Control of Human Immunodeficiency Virus Infection, But Such Responses Are Not Always Necessary for Long-Term Virus Control. J Virol (2008) 82(11):5398–407. doi: 10.1128/JVI.02176-07 PMC2395228 18353945

[36] Emu B Sinclair E Favre D Moretto WJ Hsue P Hoh R. Phenotypic, Functional, and Kinetic Parameters Associated With Apparent T-Cell Control of Human Immunodeficiency Virus Replication in Individuals With and Without Antiretroviral Treatment. J Virol (2005) 79(22):14169–78. doi: 10.1128/JVI.79.22.14169-14178.2005 PMC1280210 16254352

[37] Pereyra F Addo MM Kaufmann DE Liu Y Miura T Rathod A. Genetic and Immunologic Heterogeneity Among Persons Who Control HIV Infection in the Absence of Therapy. J Infect Dis (2008) 197(4):563–71. doi: 10.1086/526786 18275276

[38] Jiang C Lian X Gao C Sun X Einkauf KB Chevalier JM. Distinct Viral Reservoirs in Individuals With Spontaneous Control of HIV-1. Nature (2020) 585(7824):261–7. doi: 10.1038/s41586-020-2651-8 PMC7837306 32848246

[39] Madec Y Boufassa F Porter K Prins M Sabin C d’Arminio Monforte A. Natural History of HIV-Control Since Seroconversion. AIDS (2013) 27(15):2451–60. doi: 10.1097/01.aids.0000431945.72365.01 23912979

[40] Goujard C Chaix ML Lambotte O Deveau C Sinet M Guergnon J. Spontaneous Control of Viral Replication During Primary HIV Infection: When Is “HIV Controller” Status Established? Clin Infect Dis (2009) 49(6):982–6. doi: 10.1086/605504 19681706

[41] Alter G Teigen N Ahern R Streeck H Meier A Rosenberg ES. Evolution of Innate and Adaptive Effector Cell Functions During Acute HIV-1 Infection. J Infect Dis (2007) 195(10):1452–60. doi: 10.1086/513878 17436225

[42] Fellay J Shianna KV Ge D Colombo S Ledergerber B Weale M. A Whole-Genome Association Study of Major Determinants for Host Control of HIV-1. Science (2007) 317(5840):944–7. doi: 10.1126/science.1143767 PMC1991296 17641165

[43] Pereyra F Jia X McLaren PJ Telenti A de Bakker PI Walker BD. The Major Genetic Determinants of HIV-1 Control Affect Hla Class I Peptide Presentation. Science (2010) 330(6010):1551–7. doi: 10.1126/science.1195271 PMC3235490 21051598

[44] Trowsdale J Barten R Haude A Stewart CA Beck S Wilson MJ. The Genomic Context of Natural Killer Receptor Extended Gene Families. Immunol Rev (2001) 181:20–38. doi: 10.1034/j.1600-065X.2001.1810102.x 11513141

[45] Middleton D Gonzelez F. The Extensive Polymorphism of Kir Genes 1. Immunology (2010) 129(1):8–19. doi: 10.1111/j.1365-2567.2009.03208.x 20028428 PMC2807482

[46] Jiang W Johnson C Jayaraman J Simecek N Noble J Moffatt MF. Copy Number Variation Leads to Considerable Diversity for B But Not a Haplotypes of the Human Kir Genes Encoding Nk Cell Receptors. Genome Res (2012) 22(10):1845–54. doi: 10.1101/gr.137976.112 PMC3460180 22948769

[47] Kim S Sunwoo JB Yang L Choi T Song YJ French AR. Hla Alleles Determine Differences in Human Natural Killer Cell Responsiveness and Potency. Proc Natl Acad Sci USA (2008) 105(8):3053–8. doi: 10.1073/pnas.0712229105 PMC2268583 18287063

[48] Wende H Colonna M Ziegler A Volz A. Organization of the Leukocyte Receptor Cluster (Lrc) on Human Chromosome 19q13.4. Mamm Genome (1999) 10(2):154–60. doi: 10.1007/s003359900961 9922396

[49] Carrington M Norman P. The Kir Gene Cluster [Internet]. Bethesda MD, USA: National Center for Biotehnology Information US. pg 1–165. Available at: https://www.ncbi.nlm.nih.gov.

[50] Fauriat C Ivarsson MA Ljunggren HG Malmberg KJ Michaelsson J. Education of Human Natural Killer Cells by Activating Killer Cell Immunoglobulin-Like Receptors. Blood (2010) 115(6):1166–74. doi: 10.1182/blood-2009-09-245746 19903900

[51] Hsu KC Chida S Geraghty DE Dupont B. The Killer Cell Immunoglobulin-Like Receptor (Kir) Genomic Region: Gene-Order, Haplotypes and Allelic Polymorphism. Immunol Rev (2002) 190:40–52. doi: 10.1034/j.1600-065X.2002.19004.x 12493005

[52] Pyo CW Guethlein LA Vu Q Wang R Abi-Rached L Norman PJ. Different Patterns of Evolution in the Centromeric and Telomeric Regions of Group a and B Haplotypes of the Human Killer Cell Ig-Like Receptor Locus. PloS One (2010) 5(12):e15115. doi: 10.1371/journal.pone.0015115 21206914 PMC3012066

[53] Maxwell LD Wallace A Middleton D Curran MD. A Common Kir2ds4 Deletion Variant in the Human That Predicts a Soluble Kir Molecule Analogous to the Kir1d Molecule Observed in the Rhesus Monkey. Tissue Antigens (2002) 60(3):254–8. doi: 10.1034/j.1399-0039.2002.600307.x 12445308

[54] Middleton D Gonzalez A Gilmore PM. Studies on the Expression of the Deleted Kir2ds4*003 Gene Product and Distribution of Kir2ds4 Deleted and Nondeleted Versions in Different Populations. Hum Immunol (2007) 68(2):128–34. doi: 10.1016/j.humimm.2006.12.007 17321903

[55] Cella M Longo A Ferrara GB Strominger JL Colonna M. Nk3-Specific Natural Killer Cells Are Selectively Inhibited by Bw4-Positive Hla Alleles With Isoleucine 80. J Exp Med (1994) 180(4):1235–42. doi: 10.1084/jem.180.4.1235 PMC2191670 7931060

[56] Gumperz JE Litwin V Phillips JH Lanier LL Parham P. The Bw4 Public Epitope of Hla-B Molecules Confers Reactivity With Natural Killer Cell Clones That Express Nkb1, a Putative Hla Receptor. J Exp Med (1995) 181(3):1133–44. doi: 10.1084/jem.181.3.1133 PMC2191933 7532677

[57] Carr WH Pando MJ Parham P. Kir3dl1 Polymorphisms That Affect Nk Cell Inhibition by Hla-Bw4 Ligand. J Immunol (2005) 175(8):5222–9. doi: 10.4049/jimmunol.175.8.5222 16210627

[58] O’Connor DH Allen TM Vogel TU Jing P DeSouza IP Dodds E. Acute Phase Cytotoxic T Lymphocyte Escape Is a Hallmark of Simian Immunodeficiency Virus Infection. Nat Med (2002) 8(5):493–9. doi: 10.1038/nm0502-493 11984594

[59] Boudreau JE Mulrooney TJ Le Luduec JB Barker E Hsu KC. Kir3dl1 and Hla-B Density and Binding Calibrate Nk Education and Response to HIV. J Immunol (2016) 196(8):3398–410. doi: 10.4049/jimmunol.1502469 PMC4868784 26962229

[60] Anfossi N Andre P Guia S Falk CS Roetynck S Stewart CA. Human Nk Cell Education by Inhibitory Receptors for Mhc Class I. Immunity (2006) 25(2):331–42. doi: 10.1016/j.immuni.2006.06.013 16901727

[61] Trundley A Frebel H Jones D Chang C Trowsdale J. Allelic Expression Patterns of Kir3ds1 and 3dl1 Using the Z27 and Dx9 Antibodies. Eur J Immunol (2007) 37(3):780–7. doi: 10.1002/eji.200636773 17301953

[62] Biassoni R Falco M Cambiaggi A Costa P Verdiani S Pende D. Amino Acid Substitutions Can Influence the Natural Killer (Nk)-Mediated Recognition of Hla-C Molecules. Role of Serine-77 and Lysine-80 in the Target Cell Protection From Lysis Mediated by “Group 2” or “Group 1” Nk Clones. J Exp Med (1995) 182(2):605–9. doi: 10.1084/jem.182.2.605 PMC2192139 7629517

[63] Colonna M Borsellino G Falco M Ferrara GB Strominger JL. Hla-C Is the Inhibitory Ligand That Determines Dominant Resistance to Lysis by Nk1- and Nk2-Specific Natural Killer Cells. Proc Natl Acad Sci USA (1993) 90(24):12000–4. doi: 10.1073/pnas.90.24.12000 PMC48113 8265660

[64] Moesta AK Norman PJ Yawata M Yawata N Gleimer M Parham P. Synergistic Polymorphism at Two Positions Distal to the Ligand-Binding Site Makes Kir2dl2 a Stronger Receptor for Hla-C Than Kir2dl3. J Immunol (2008) 180(6):3969–79. doi: 10.4049/jimmunol.180.6.3969 18322206

[65] Winter CC Gumperz JE Parham P Long EO Wagtmann N. Direct Binding and Functional Transfer of Nk Cell Inhibitory Receptors Reveal Novel Patterns of Hla-C Allotype Recognition. J Immunol (1998) 161(2):571–7. 9670929

[66] Braud VM Allan DS O’Callaghan CA Soderstrom K D’Andrea A Ogg GS. Hla-E Binds to Natural Killer Cell Receptors Cd94/Nkg2a, B and C. Nature (1998) 391(6669):795–9. doi: 10.1038/35869 9486650

[67] Lee N Llano M Carretero M Ishitani A Navarro F Lopez-Botet M. Hla-E Is a Major Ligand for the Natural Killer Inhibitory Receptor Cd94/Nkg2a. Proc Natl Acad Sci USA (1998) 95(9):5199–204. doi: 10.1073/pnas.95.9.5199 PMC20238 9560253

[68] Llano M Lee N Navarro F Garcia P Albar JP Geraghty DE. Hla-E-Bound Peptides Influence Recognition by Inhibitory and Triggering Cd94/Nkg2 Receptors: Preferential Response to an Hla-G-Derived Nonamer. Eur J Immunol (1998) 28(9):2854–63. doi: 10.1002/(SICI)1521-4141(199809)28:09<2854::AID-IMMU2854>3.0.CO;2-W 9754572

[69] Yawata M Yawata N Draghi M Partheniou F Little AM Parham P. Mhc Class I-Specific Inhibitory Receptors and Their Ligands Structure Diverse Human Nk-Cell Repertoires Toward a Balance of Missing Self-Response. Blood (2008) 112(6):2369–80. doi: 10.1182/blood-2008-03-143727 PMC2532809 18583565

[70] Wu N Veillette A. Slam Family Receptors in Normal Immunity and Immune Pathologies. Curr Opin Immunol (2016) 38:45–51. doi: 10.1016/j.coi.2015.11.003 26682762

[71] Long EO. Regulation of Immune Responses Through Inhibitory Receptors. Annu Rev Immunol (1999) 17:875–904. doi: 10.1146/annurev.immunol.17.1.875 10358776

[72] Watzl C Stebbins CC Long EO. Nk Cell Inhibitory Receptors Prevent Tyrosine Phosphorylation of the Activation Receptor 2b4 (Cd244). J Immunol (2000) 165(7):3545–8. doi: 10.4049/jimmunol.165.7.3545 11034353

[73] Elliott JM Yokoyama WM. Unifying Concepts of Mhc-Dependent Natural Killer Cell Education. Trends Immunol (2011) 32(8):364–72. doi: 10.1016/j.it.2011.06.001 PMC3151350 21752715

[74] Joncker NT Fernandez NC Treiner E Vivier E Raulet DH. Nk Cell Responsiveness Is Tuned Commensurate With the Number of Inhibitory Receptors for Self-Mhc Class I: The Rheostat Model. J Immunol (2009) 182(8):4572–80. doi: 10.4049/jimmunol.0803900 PMC2938179 19342631

[75] Yu J Heller G Chewning J Kim S Yokoyama WM Hsu KC. Hierarchy of the Human Natural Killer Cell Response Is Determined by Class and Quantity of Inhibitory Receptors for Self-Hla-B and Hla-C Ligands. J Immunol (2007) 179(9):5977–89. doi: 10.4049/jimmunol.179.9.5977 17947671

[76] Fernandez NC Lozier A Flament C Ricciardi-Castagnoli P Bellet D Suter M. Dendritic Cells Directly Trigger Nk Cell Functions: Cross-Talk Relevant in Innate Anti-Tumor Immune Responses *In Vivo* . Nat Med (1999) 5(4):405–11. doi: 10.1038/7403 10202929

[77] Qi Y Martin MP Gao X Jacobson L Goedert JJ Buchbinder S. Kir/Hla Pleiotropism: Protection Against Both HIV and Opportunistic Infections. PloS Pathog (2006) 2(8):0741–5. doi: 10.1371/journal.ppat.0020079 PMC1550271 16933987

[78] Migueles SA Sabbaghian MS Shupert WL Bettinotti MP Marincola FM Martino L. Hla B*5701 Is Highly Associated With Restriction of Virus Replication in a Subgroup of HIV-Infected Long Term Nonprogressors. Proc Natl Acad Sci USA (2000) 97(6):2709–14. doi: 10.1073/pnas.050567397 PMC15994 10694578

[79] Kaslow RA Carrington M Apple R Park L Munoz A Saah AJ. Influence of Combinations of Human Major Histocompatibility Complex Genes on the Course of HIV-1 Infection. Nat Med (1996) 2(4):405–11. doi: 10.1038/nm0496-405 8597949

[80] Leslie AJ Pfafferott KJ Chetty P Draenert R Addo MM Feeney M. HIV Evolution: Ctl Escape Mutation and Reversion After Transmission. Nat Med (2004) 10(3):282–9. doi: 10.1038/nm992 14770175

[81] Gao X Bashirova A Iversen AK Phair J Goedert JJ Buchbinder S. Aids Restriction Hla Allotypes Target Distinct Intervals of HIV-1 Pathogenesis. Nat Med (2005) 11(12):1290–2. doi: 10.1038/nm1333 16288280

[82] Martin MP Naranbhai V Shea PR Qi Y Ramsuran V Vince N. Killer Cell Immunoglobulin-Like Receptor 3dl1 Variation Modifies Hla-B*57 Protection Against HIV-1. J Clin Invest (2018) 128(5):1903–12. doi: 10.1172/JCI98463 PMC5919796 29461980

[83] Corrah TW Goonetilleke N Kopycinski J Deeks SG Cohen MS Borrow P. Reappraisal of the Relationship Between the HIV-1-Protective Single-Nucleotide Polymorphism 35 Kilobases Upstream of the Hla-C Gene and Surface Hla-C Expression. J Virol (2011) 85(7):3367–74. doi: 10.1128/JVI.02276-10 PMC3067890 21248048

[84] Kulpa DA Collins KL. The Emerging Role of Hla-C in HIV-1 Infection. Immunology (2011) 134(2):116–22. doi: 10.1111/j.1365-2567.2011.03474.x PMC3194220 21896007

[85] Zipeto D Beretta A. Hla-C and HIV-1: Friends or Foes? Retrovirology (2012) 9:39. doi: 10.1186/1742-4690-9-39 22571741 PMC3386009

[86] Fellay J Ge D Shianna KV Colombo S Ledergerber B Cirulli ET. Common Genetic Variation and the Control of HIV-1 in Humans. PloS Genet (2009) 5(12):e1000791. doi: 10.1371/journal.pgen.1000791 20041166 PMC2791220

[87] Thomas R Apps R Qi Y Gao X Male V O’hUigin C. Hla-C Cell Surface Expression and Control of HIV/Aids Correlate With a Variant Upstream of Hla-C. Nat Genet (2009) 41(12):1290–4. doi: 10.1038/ng.486 PMC2887091 19935663

[88] Kulkarni S Savan R Qi Y Gao X Yuki Y Bass SE. Differential Microrna Regulation of Hla-C Expression and Its Association With HIV Control. Nature (2011) 472(7344):495–8. doi: 10.1038/nature09914 PMC3084326 21499264

[89] Blais ME Dong T Rowland-Jones S. Hla-C as a Mediator of Natural Killer and T-Cell Activation: Spectator or Key Player? Immunology (2011) 133(1):1–7. doi: 10.1111/j.1365-2567.2011.03422.x 21355865 PMC3088962

[90] Apps R Qi Y Carlson JM Chen H Gao X Thomas R. Influence of Hla-C Expression Level on HIV Control. Science (2013) 340(6128):87–91. doi: 10.1126/science.1232685 23559252 PMC3784322

[91] Malnati MS Ugolotti E Monti MC Battista D Vanni I Bordo D. Activating Killer Immunoglobulin Receptors and Hla-C: A Successful Combination Providing HIV-1 Control. Sci Rep (2017) 7:42470. doi: 10.1038/srep42470 28211903 PMC5304173

[92] Braud V Jones EY McMichael A. The Human Major Histocompatibility Complex Class Ib Molecule Hla-E Binds Signal Sequence-Derived Peptides With Primary Anchor Residues at Positions 2 and 9. Eur J Immunol (1997) 27(5):1164–9. doi: 10.1002/eji.1830270517 9174606

[93] Lee N Goodlett DR Ishitani A Marquardt H Geraghty DE. Hla-E Surface Expression Depends on Binding of Tap-Dependent Peptides Derived From Certain Hla Class I Signal Sequences. J Immunol (1998) 160(10):4951–60. 9590243

[94] Horowitz A Djaoud Z Nemat-Gorgani N Blokhuis J Hilton HG Beziat V. Class I Hla Haplotypes Form Two Schools That Educate Nk Cells in Different Ways. Sci Immunol (2016) 1(3). doi: 10.1126/sciimmunol.aag1672 PMC5110269 27868107

[95] Ramsuran V Naranbhai V Horowitz A Qi Y Martin MP Yuki Y. Elevated Hla-A Expression Impairs HIV Control Through Inhibition of Nkg2a-Expressing Cells. Science (2018) 359(6371):86–90. doi: 10.1126/science.aam8825 29302013 PMC5933048

[96] Carlson JM Listgarten J Pfeifer N Tan V Kadie C Walker BD. Widespread Impact of Hla Restriction on Immune Control and Escape Pathways of HIV-1. J Virol (2012) 86(9):5230–43. doi: 10.1128/JVI.06728-11 PMC3347390 22379086

[97] Leslie A Matthews PC Listgarten J Carlson JM Kadie C Ndung’u T. Additive Contribution of Hla Class I Alleles in the Immune Control of HIV-1 Infection. J Virol (2010) 84(19):9879–88. doi: 10.1128/JVI.00320-10 PMC2937780 20660184

[98] Vieira VA Adland E Malone DFG Martin MP Groll A Ansari MA. An Hla-I Signature Favouring Kir-Educated Natural Killer Cells Mediates Immune Control of HIV in Children and Contrasts With the Hla-B-Restricted Cd8+ T-Cell-Mediated Immune Control in Adults. PloS Pathog (2021) 17(11):e1010090. doi: 10.1371/journal.ppat.1010090 34793581 PMC8639058

[99] Singh KK Qin M Brummel SS Angelidou K Trout RN Fenton T. Killer Cell Immunoglobulin-Like Receptor Alleles Alter HIV Disease in Children. PloS One (2016) 11(3):e0151364. doi: 10.1371/journal.pone.0151364 26983081 PMC4794224

[100] Boulet S Song R Kamya P Bruneau J Shoukry NH Tsoukas CM. HIV Protective Kir3dl1 and Hla-B Genotypes Influence Nk Cell Function Following Stimulation With Hla-Devoid Cells. J Immunol (2010) 184(4):2057–64. doi: 10.4049/jimmunol.0902621 20061407

[101] Kamya P Boulet S Tsoukas CM Routy JP Thomas R Cote P. Receptor-Ligand Requirements for Increased Nk Cell Poly-Functional Potential in *H/*Y+B57 HIV-1 Infected Slow Progressors. J Virol (2011) 85(12):5949–60. doi: 10.1128/JVI.02652-10 PMC3126301 21471235

[102] Alter G Teigen N Davis BT Addo MM Suscovich TJ Waring MT. Sequential Deregulation of Nk Cell Subset Distribution and Function Starting in Acute HIV-1 Infection. Blood (2005) 106(10):3366–9. doi: 10.1182/blood-2005-03-1100 16002429

[103] Mavilio D Lombardo G Benjamin J Kim D Follman D Marcenaro E. Characterization of Cd56-/Cd16+ Natural Killer (Nk) Cells: A Highly Dysfunctional Nk Subset Expanded in HIV-Infected Viremic Individuals. Proc Natl Acad Sci USA (2005) 102(8):2886–91. doi: 10.1073/pnas.0409872102 PMC549494 15699323

[104] O’Connor GM Holmes A Mulcahy F Gardiner CM. Natural Killer Cells From Long-Term Non-Progressor HIV Patients Are Characterized by Altered Phenotype and Function. Clin Immunol (2007) 124(3):277–83. doi: 10.1016/j.clim.2007.05.016 17611161

[105] Parsons MS Wren L Isitman G Navis M Stratov I Bernard NF. HIV Infection Abrogates the Functional Advantage of Natural Killer Cells Educated Through Kir3dl1/Hla-Bw4 Interactions to Mediate Anti-HIV Antibody-Dependent Cellular Cytotoxicity. J Virol (2012) 86(8):4488–95. doi: 10.1128/JVI.06112-11 PMC3318670 22345455

[106] Vieillard V Fausther-Bovendo H Samri A Debre P. Specific Phenotypic and Functional Features of Natural Killer Cells From HIV-Infected Long-Term Nonprogressors and HIV Controllers. J Acquir Immune Defic Syndr (2010) 53(5):564–73. doi: 10.1097/QAI.0b013e3181d0c5b4 20147841

[107] Bernard NF Melendez-Pena C Kamya P Tsoukas CM Boulassel M-R Routy J-P. Natural Killer Cells From HIV Infected Slow Progressors Who Carry the Protective Hla-B* 27 Allele and Inhibitory Kir3dl1 Receptors Have Elevated Poly-Functional Potential Compared to Bw6 Homozygotes. In: Aghdassi DA, editor. HIV Infection in the Era of Highly Active Antiretroviral Treatment and Some of Its Associated Complications. Rijeka, Croatia: InTech (2011).

[108] Parsons MS Boulet S Song R Bruneau J Shoukry NH Routy JP. Mind the Gap: Lack of Association Between Kir3dl1*004/Hla-Bw4-Induced Natural Killer Cell Function and Protection From HIV Infection. J Infect Dis (2010) 202(Suppl 3):S356–S60. doi: 10.1086/655966 20887224

[109] Pando MJ Gardiner CM Gleimer M McQueen KL Parham P. The Protein Made From a Common Allele of Kir3dl1 (3dl1*004) Is Poorly Expressed at Cell Surfaces Due to Substitution at Positions 86 in Ig Domain 0 and 182 in Ig Domain 1. J Immunol (2003) 171(12):6640–9. doi: 10.4049/jimmunol.171.12.6640 14662867

[110] Taner SB Pando MJ Roberts A Schellekens J Marsh SG Malmberg KJ. Interactions of Nk Cell Receptor Kir3dl1*004 With Chaperones and Conformation-Specific Antibody Reveal a Functional Folded State as Well as Predominant Intracellular Retention. J Immunol (2011) 186(1):62–72. doi: 10.4049/jimmunol.0903657 21115737 PMC3129036

[111] Korner C Simoneau CR Schommers P Granoff M Ziegler M Holzemer A. HIV-1-Mediated Downmodulation of Hla-C Impacts Target Cell Recognition and Antiviral Activity of Nk Cells. Cell Host Microbe (2017) 22(1):111–9 e4. doi: 10.1016/j.chom.2017.06.008 28704647 PMC5565794

[112] Cohen GB Gandhi RT Davis DM Mandelboim O Chen BK Strominger JL. The Selective Downregulation of Class I Major Histocompatibility Complex Proteins by HIV-1 Protects HIV-Infected Cells From Nk Cells. Immunity (1999) 10(6):661–71. doi: 10.1016/S1074-7613(00)80065-5 10403641

[113] Bonaparte MI Barker E. Killing of Human Immunodeficiency Virus-Infected Primary T-Cell Blasts by Autologous Natural Killer Cells Is Dependent on the Ability of the Virus to Alter the Expression of Major Histocompatibility Complex Class I Molecules. Blood (2004) 104(7):2087–94. doi: 10.1182/blood-2004-02-0696 15117765

[114] Apps R Del Prete GQ Chatterjee P Lara A Brumme ZL Brockman MA. HIV-1 Vpu Mediates Hla-C Downregulation. Cell Host Microbe (2016) 19(5):686–95. doi: 10.1016/j.chom.2016.04.005 PMC4904791 27173934

[115] Collins KL Chen BK Kalams SA Walker BD Baltimore D. HIV-1 Nef Protein Protects Infected Primary Cells Against Killing by Cytotoxic T Lymphocytes. Nature (1998) 391(6665):397–401. doi: 10.1038/34929 9450757

[116] Schwartz O Marechal V Le Gall S Lemonnier F Heard JM. Endocytosis of Major Histocompatibility Complex Class I Molecules Is Induced by the HIV-1 Nef Protein. Nat Med (1996) 2(3):338–42. doi: 10.1038/nm0396-338 8612235

[117] Kiani Z Dupuy FP Bruneau J Lebouche B Retiere C Geraghty DE. The Education of Nk Cells Determines Their Responsiveness to Autologous HIV-Infected Cd4 T Cells. J Virol (2019) 93(23):1–14. doi: 10.1128/JVI.01185-19 PMC6854491 31511383

[118] Song R Lisovsky I Lebouche B Routy JP Bruneau J Bernard NF. HIV Protective Kir3dl1/S1-Hla-B Genotypes Influence Nk Cell-Mediated Inhibition of HIV Replication in Autologous Cd4 Targets. PloS Pathog (2014) 10(1):e1003867. doi: 10.1371/journal.ppat.1003867 24453969 PMC3894215

[119] Oliva A Kinter AL Vaccarezza M Rubbert A Catanzaro A Moir S. Natural Killer Cells From Human Immunodeficiency Virus (HIV)-Infected Individuals Are an Important Source of Cc-Chemokines and Suppress HIV-1 Entry and Replication *In Vitro* . J Clin Invest (1998) 102(1):223–31. doi: 10.1172/JCI2323 PMC509084 9649576

[120] Alter G Martin MP Teigen N Carr WH Suscovich TJ Schneidewind A. Differential Natural Killer Cell-Mediated Inhibition of HIV-1 Replication Based on Distinct Kir/Hla Subtypes. J Exp Med (2007) 204(12):3027–36. doi: 10.1084/jem.20070695 PMC2118524 18025129

[121] Garcia-Beltran WF Holzemer A Martrus G Chung AW Pacheco Y Simoneau CR. Open Conformers of Hla-F Are High-Affinity Ligands of the Activating Nk-Cell Receptor Kir3ds1. Nat Immunol (2016) 17(9):1067–74. doi: 10.1038/ni.3513 PMC4992421 27455421

[122] Gillespie GM Bashirova A Dong T McVicar DW Rowland-Jones SL Carrington M. Lack of Kir3ds1 Binding to Mhc Class I Bw4 Tetramers in Complex With Cd8+ T Cell Epitopes. AIDS Res Hum Retroviruses (2007) 23(3):451–5. doi: 10.1089/aid.2006.0165 17411378

[123] Tallon BJ Bruneau J Tsoukas CM Routy JP Kiani Z Tan X. Time to Seroconversion in HIV-Exposed Subjects Carrying Protective Versus Non Protective Kir3ds1/L1 and Hla-B Genotypes. PloS One (2014) 9(10):e110480. doi: 10.1371/journal.pone.0110480 25330014 PMC4201542

[124] O’Connor GM Vivian JP Gostick E Pymm P Lafont BA Price DA. Peptide-Dependent Recognition of Hla-B*57:01 by Kir3ds1. J Virol (2015) 89(10):5213–21. doi: 10.1128/JVI.03586-14 PMC4442525 25740999

[125] Kiani Z Dupuy FP Bruneau J Lebouche B Zhang CX Jackson E. Hla-F on Hla-Null 721.221 Cells Activates Primary Nk Cells Expressing the Activating Killer Ig-Like Receptor Kir3ds1. J Immunol (2018) 201(1):113–23. doi: 10.4049/jimmunol.1701370 29743316

[126] Kiani Z Bruneau J Geraghty DE F BN. Hla-F on Autologous HIV-Infected Cells Activates Primary Nk Cells Expressing the Activating Killer Immunoglobulin-Like Receptor Kir3ds1. J Virol (2019) 93(18):1–14. doi: 10.1128/JVI PMC6714807 31270222

[127] Maldarelli F. The Role of HIV Integration in Viral Persistence: No More Whistling Past the Proviral Graveyard. J Clin Invest (2016) 126(2):438–47. doi: 10.1172/JCI80564 PMC4731194 26829624

[128] Marras F Casabianca A Bozzano F Ascierto ML Orlandi C Di Biagio A. Control of the HIV-1 DNA Reservoir Is Associated *In Vivo* and *In Vitro* With Nkp46/Nkp30 (Cd335 Cd337) Inducibility and Interferon Gamma Production by Transcriptionally Unique Nk Cells. J Virol (2017) 91(23):1–19. doi: 10.1128/JVI.00647-17 PMC5686752 28956765

[129] Pohlmeyer CW Gonzalez VD Irrinki A Ramirez RN Li L Mulato A. Identification of Nk Cell Subpopulations That Differentiate HIV-Infected Subject Cohorts With Diverse Levels of Virus Control. J Virol (2019) 93(7):1–15. doi: 10.1128/JVI.01790-18 PMC6430550 30700608

[130] Bruhns P Iannascoli B England P Mancardi DA Fernandez N Jorieux S. Specificity and Affinity of Human Fcgamma Receptors and Their Polymorphic Variants for Human Igg Subclasses. Blood (2009) 113(16):3716–25. doi: 10.1182/blood-2008-09-179754 19018092

[131] Shields RL Lai J Keck R O’Connell LY Hong K Meng YG. Lack of Fucose on Human Igg1 N-Linked Oligosaccharide Improves Binding to Human Fcgamma Riii and Antibody-Dependent Cellular Toxicity. J Biol Chem (2002) 277(30):26733–40. doi: 10.1074/jbc.M202069200 11986321

[132] Anand SP Ding S Tolbert WD Prevost J Richard J Gil HM. Enhanced Ability of Plant-Derived Pgt121 Glycovariants to Eliminate HIV-1-Infected Cells. J Virol (2021) 95(18):e0079621. doi: 10.1128/JVI.00796-21 34232070 PMC8387047

[133] Ravetch JV Perussia B. Alternative Membrane Forms of Fc Gamma Riii(Cd16) on Human Natural Killer Cells and Neutrophils. Cell Type-Specific Expression of Two Genes That Differ in Single Nucleotide Substitutions. J Exp Med (1989) 170(2):481–97. doi: 10.1084/jem.170.2.481 PMC2189395 2526846

[134] Wu J Edberg JC Redecha PB Bansal V Guyre PM Coleman K. A Novel Polymorphism of Fcgammariiia (Cd16) Alters Receptor Function and Predisposes to Autoimmune Disease. J Clin Invest (1997) 100(5):1059–70. doi: 10.1172/JCI119616 PMC508280 9276722

[135] Weng WK Levy R. Two Immunoglobulin G Fragment C Receptor Polymorphisms Independently Predict Response to Rituximab in Patients With Follicular Lymphoma. J Clin Oncol (2003) 21(21):3940–7. doi: 10.1200/JCO.2003.05.013 12975461

[136] Checkley MA Luttge BG Freed EO. HIV-1 Envelope Glycoprotein Biosynthesis, Trafficking, and Incorporation. J Mol Biol (2011) 410(4):582–608. doi: 10.1016/j.jmb.2011.04.042 21762802 PMC3139147

[137] Alsulami K Bolastig N Dupuy FP Mabanga T Gilbert L Kiani Z. Influence of Nkg2c Genotypes on HIV Susceptibility and Viral Load Set Point. J Virol (2021) 95(16):e0041721. doi: 10.1128/JVI.00417-21 34076484 PMC8312870

[138] Kant S Zhang N Barbe A Routy JP Tremblay C Thomas R. Polyfunctional Fc Dependent Activity of Antibodies to Native Trimeric Envelope in HIV Elite Controllers. Front Immunol (2020) 11:583820. doi: 10.3389/fimmu.2020.583820 33101312 PMC7555699

[139] Richard J Veillette M Batraville LA Coutu M Chapleau JP Bonsignori M. Flow Cytometry-Based Assay to Study HIV-1 Gp120 Specific Antibody-Dependent Cellular Cytotoxicity Responses. J Virol Methods (2014) 208:107–14. doi: 10.1016/j.jviromet.2014.08.003 25125129

[140] Veillette M Desormeaux A Medjahed H Gharsallah NE Coutu M Baalwa J. Interaction With Cellular Cd4 Exposes HIV-1 Envelope Epitopes Targeted by Antibody-Dependent Cell-Mediated Cytotoxicity. J Virol (2014) 88(5):2633–44. doi: 10.1128/JVI.03230-13 PMC3958102 24352444

[141] Yates NL Liao HX Fong Y DeCamp A Vandergrift NA Williams WT. Vaccine-Induced Env V1-V2 Igg3 Correlates With Lower HIV-1 Infection Risk and Declines Soon After Vaccination. Sci Transl Med (2014) 6(228):228ra39. doi: 10.1126/scitranslmed.3007730 PMC4116665 24648342

[142] Ackerman ME Mikhailova A Brown EP Dowell KG Walker BD Bailey-Kellogg C. Polyfunctional HIV-Specific Antibody Responses Are Associated With Spontaneous HIV Control. PloS Pathog (2016) 12(1):e1005315. doi: 10.1371/journal.ppat.1005315 26745376 PMC4706315

[143] Gómez-Román VR Florese RH Peng B Montefiori DC Kalyanaraman VS Venzon D. An Adenovirus-Based HIV Subtype B Prime/Boost Vaccine Regimen Elicits Antibodies Mediating Broad Antibody-Dependent Cellular Cytotoxicity Against Non-Subtype B HIV Strains. J Acquir Immune Defic Syndr (2006) 43(3):270–7. doi: 10.1097/01.qai.0000230318.40170.60 16940858

[144] Guan Y Sajadi MM Kamin-Lewis R Fouts TR Dimitrov A Zhang Z. Discordant Memory B Cell and Circulating Anti-Env Antibody Responses in HIV-1 Infection. Proc Natl Acad Sci (2009) 106(10):3952–7. doi: 10.1073/pnas.0813392106 PMC2644653 19225108

[145] Milligan C Richardson BA John-Stewart G Nduati R Overbaugh J. Passively Acquired Antibody-Dependent Cellular Cytotoxicity (Adcc) Activity in HIV-Infected Infants Is Associated With Reduced Mortality. Cell Host Microbe (2015) 17(4):500–6. doi: 10.1016/j.chom.2015.03.002 PMC4392343 25856755

[146] Lambotte O Ferrari G Moog C Yates NL Liao HX Parks RJ. Heterogeneous Neutralizing Antibody and Antibody-Dependent Cell Cytotoxicity Responses in HIV-1 Elite Controllers. AIDS (2009) 23(8):897–906. doi: 10.1097/QAD.0b013e328329f97d 19414990 PMC3652655

[147] Madhavi V Wines BD Amin J Emery S Group ES Lopez E. HIV-1 Env- and Vpu-Specific Antibody-Dependent Cellular Cytotoxicity Responses Associated With Elite Control of HIV. J Virol (2017) 91(18):e00700–17. doi: 10.1128/JVI.00700-17 PMC5571238 28701393

[148] Williams KL Cortez V Dingens AS Gach JS Rainwater S Weis JF. HIV-Specific Cd4-Induced Antibodies Mediate Broad and Potent Antibody-Dependent Cellular Cytotoxicity Activity and Are Commonly Detected in Plasma From HIV-Infected Humans. EBioMedicine (2015) 2(10):1464–77. doi: 10.1016/j.ebiom.2015.09.001 PMC4634620 26629541

[149] Trkola A Matthews J Gordon C Ketas T Moore JP. A Cell Line-Based Neutralization Assay for Primary Human Immunodeficiency Virus Type 1 Isolates That Use Either the Ccr5 or the Cxcr4 Coreceptor. J Virol (1999) 73(11):8966–74. doi: 10.1128/JVI.73.11.8966-8974.1999 PMC112928 10516002

[150] Trkola A Dragic T Arthos J Binley JM Olson WC Allaway GP. Cd4-Dependent, Antibody-Sensitive Interactions Between HIV-1 and Its Co-Receptor Ccr-5. Nature (1996) 384(6605):184–7. doi: 10.1038/384184a0 8906796

[151] Howell DN Andreotti PE Dawson JR Cresswell P. Natural Killing Target Antigens as Inducers of Interferon: Studies With an Immunoselected, Natural Killing-Resistant Human T Lymphoblastoid Cell Line. J Immunol (1985) 134(2):971–6. 3871222

[152] von Bredow B Arias JF Heyer LN Moldt B Le K Robinson JE. Comparison of Antibody-Dependent Cell-Mediated Cytotoxicity and Virus Neutralization by HIV-1 Env-Specific Monoclonal Antibodies. J Virol (2016) 90(13):6127–39. doi: 10.1128/JVI.00347-16 PMC4907221 27122574

[153] Bruel T Guivel-Benhassine F Lorin V Lortat-Jacob H Baleux F Bourdic K. Lack of Adcc Breadth of Human Nonneutralizing Anti-HIV-1 Antibodies. J Virol (2017) 91(8):1–19. doi: 10.1128/JVI.02440-16 PMC5375671 28122982

[154] Bruel T Guivel-Benhassine F Amraoui S Malbec M Richard L Bourdic K. Elimination of HIV-1-Infected Cells by Broadly Neutralizing Antibodies. Nat Commun (2016) 7:10844. doi: 10.1038/ncomms10844 26936020 PMC4782064

[155] Dupuy FP Kant S Barbe A Routy JP Bruneau J Lebouche B. Antibody-Dependent Cellular Cytotoxicity-Competent Antibodies Against HIV-1-Infected Cells in Plasma From HIV-Infected Subjects. mBio (2019) 10(6):1–21. doi: 10.1128/mBio.02690-19 PMC6918083 31848282

[156] Richard J Prevost J Baxter AE von Bredow B Ding S Medjahed H. Uninfected Bystander Cells Impact the Measurement of HIV-Specific Antibody-Dependent Cellular Cytotoxicity Responses. mBio (2018) 9(2):1–19. doi: 10.1128/mBio.00358-18 PMC5874913 29559570

[157] Ding S Veillette M Coutu M Prevost J Scharf L Bjorkman PJ. A Highly Conserved Residue of the HIV-1 Gp120 Inner Domain Is Important for Antibody-Dependent Cellular Cytotoxicity Responses Mediated by Anti-Cluster a Antibodies. J Virol (2015) 90(4):2127–34. doi: 10.1128/JVI.02779-15 PMC4733974 26637462

[158] Richard J Prevost J Alsahafi N Ding S Finzi A. Impact of HIV-1 Envelope Conformation on Adcc Responses. Trends Microbiol (2018) 26(4):253–65. doi: 10.1016/j.tim.2017.10.007 29162391

[159] Magadan JG Perez-Victoria FJ Sougrat R Ye Y Strebel K Bonifacino JS. Multilayered Mechanism of Cd4 Downregulation by HIV-1 Vpu Involving Distinct Er Retention and Erad Targeting Steps. PloS Pathog (2010) 6(4):e1000869. doi: 10.1371/journal.ppat.1000869 20442859 PMC2861688

[160] Nagel L Kant S Leeks C Routy JP Tremblay C Thomas R. Evolution of Antibodies to Native Trimeric Envelope and Their Fc-Dependent Functions in Untreated and Treated Primary HIV Infection. J Virol (2021) 95(24):e0162521. doi: 10.1128/JVI.01625-21 34586863 PMC8610575

[161] Gomez-Roman VR Florese RH Patterson LJ Peng B Venzon D Aldrich K. A Simplified Method for the Rapid Fluorometric Assessment of Antibody-Dependent Cell-Mediated Cytotoxicity. J Immunol Methods (2006) 308(1-2):53–67. doi: 10.1016/j.jim.2005.09.018 16343526

[162] Kramski M Schorcht A Johnston AP Lichtfuss GF Jegaskanda S De Rose R. Role of Monocytes in Mediating HIV-Specific Antibody-Dependent Cellular Cytotoxicity. J Immunol Methods (2012) 384(1-2):51–61. doi: 10.1016/j.jim.2012.07.006 22841577

[163] Pollara J Hart L Brewer F Pickeral J Packard BZ Hoxie JA. High-Throughput Quantitative Analysis of HIV-1 and Siv-Specific Adcc-Mediating Antibody Responses. Cytometry A (2011) 79(8):603–12. doi: 10.1002/cyto.a.21084 PMC3692008 21735545

[164] Richard J Prevost J von Bredow B Ding S Brassard N Medjahed H. Bst-2 Expression Modulates Small Cd4-Mimetic Sensitization of HIV-1-Infected Cells to Antibody-Dependent Cellular Cytotoxicity. J Virol (2017) 91(11):1–13. doi: 10.1128/JVI.00219-17 PMC5432882 28331088

[165] Edmonds TG Ding H Yuan X Wei Q Smith KS Conway JA. Replication Competent Molecular Clones of HIV-1 Expressing Renilla Luciferase Facilitate the Analysis of Antibody Inhibition in Pbmc. Virology (2010) 408(1):1–13. doi: 10.1016/j.virol.2010.08.028 20863545 PMC2993081

[166] Alberti MO Jones JJ Miglietta R Ding H Bakshi RK Edmonds TG. Optimized Replicating Renilla Luciferase Reporter HIV-1 Utilizing Novel Internal Ribosome Entry Site Elements for Native Nef Expression and Function. AIDS Res Hum Retroviruses (2015) 31(12):1278–96. doi: 10.1089/aid.2015.0074 PMC4663642 26101895

[167] Huang Y Ferrari G Alter G Forthal DN Kappes JC Lewis GK. Diversity of Antiviral Igg Effector Activities Observed in HIV-Infected and Vaccinated Subjects. J Immunol (2016) 197(12):4603–12. doi: 10.4049/jimmunol.1601197 PMC5137799 27913647

[168] Chung AW Navis M Isitman G Wren L Silvers J Amin J. Activation of Nk Cells by Adcc Antibodies and HIV Disease Progression. J Acquir Immune Defic Syndr (2011) 58(2):127–31. doi: 10.1097/QAI.0b013e31822c62b9 PMC3175260 21792067

[169] Lambotte O Pollara J Boufassa F Moog C Venet A Haynes BF. High Antibody-Dependent Cellular Cytotoxicity Responses Are Correlated With Strong Cd8 T Cell Viral Suppressive Activity But Not With B57 Status in HIV-1 Elite Controllers. PloS One (2013) 8(9):e74855. doi: 10.1371/journal.pone.0074855 24086385 PMC3781132

[170] Kant S Zhang N Routy JP Tremblay C Thomas R Szabo J. Quantifying Anti-HIV Envelope-Specific Antibodies in Plasma From HIV Infected Individuals. Viruses (2019) 11(6). doi: 10.3390/v11060487 PMC6631318 31141927

[171] Ahmad R Sindhu ST Toma E Morisset R Vincelette J Menezes J. Evidence for a Correlation Between Antibody-Dependent Cellular Cytotoxicity-Mediating Anti-HIV-1 Antibodies and Prognostic Predictors of HIV Infection. J Clin Immunol (2001) 21(3):227–33. doi: 10.1023/A:1011087132180 11403230

[172] Gondois-Rey F Cheret A Granjeaud S Mallet F Bidaut G Lecuroux C. Nkg2c+ Memory-Like Nk Cells Contribute to the Control of HIV Viremia During Primary Infection: Optiprim-Anrs 147. Clin Transl Immunol (2017) 6(7):e150. doi: 10.1038/cti.2017.22 PMC5539415 28791125

[173] Chen X Lin M Qian S Zhang Z Fu Y Xu J. The Early Antibody-Dependent Cell-Mediated Cytotoxicity Response Is Associated With Lower Viral Set Point in Individuals With Primary HIV Infection. Front Immunol (2018) 9:2322. doi: 10.3389/fimmu.2018.02322 30356637 PMC6189277

[174] Tiemessen CT Shalekoff S Meddows-Taylor S Schramm DB Papathanasopoulos M Gray G. Natural Killer Cells That Respond to Human Immunodeficiency Virus Type 1 (HIV-1) Peptides Are Associated With Control of HIV-1 Infection. J Infect Dis (2010) 202(9):1444–53. doi: 10.1086/656535 PMC3060333 20874516

[175] Alter G Dowell KG Brown EP Suscovich TJ Mikhailova A Mahan AE. High-Resolution Definition of Humoral Immune Response Correlates of Effective Immunity Against HIV. Mol Syst Biol (2018) 14(3):e7881. doi: 10.15252/msb.20177881 29581149 PMC5868198

[176] Lisovsky I Kant S Tremblay-McLean A Isitman G Kiani Z Dupuy FP. Differential Contribution of Education Through Kir2dl1, Kir2dl3, and Kir3dl1 to Antibody-Dependent (Ad) Nk Cell Activation and Adcc. J Leukoc Biol (2019) 105(3):551–63. doi: 10.1002/JLB.4A0617-242RRR PMC6916277 30698860

[177] Isitman G Lisovsky I Tremblay-McLean A Parsons MS Shoukry NH Wainberg MA. Natural Killer Cell Education Does Not Affect the Magnitude of Granzyme B Delivery to Target Cells by Antibody-Dependent Cellular Cytotoxicity. AIDS (2015) 29(12):1433–43. doi: 10.1097/QAD.0000000000000729 26244383

[178] Bernard NF Kiani Z Tremblay-McLean A Kant SA Leeks CE Dupuy FP. Natural Killer (Nk) Cell Education Differentially Influences HIV Antibody-Dependent Nk Cell Activation and Antibody-Dependent Cellular Cytotoxicity. Front Immunol (2017) 8:1033. doi: 10.3389/fimmu.2017.01033 28883824 PMC5574056

[179] Lopez-Verges S Milush JM Schwartz BS Pando MJ Jarjoura J York VA. Expansion of a Unique Cd57(+)Nkg2chi Natural Killer Cell Subset During Acute Human Cytomegalovirus Infection. Proc Natl Acad Sci USA (2011) 108(36):14725–32. doi: 10.1073/pnas.1110900108 PMC3169160 21825173

[180] Chang C Rodríguez A Carretero M López-Botet M Phillips JH Lanier LL. Molecular Characterization of Human Cd94: A Type Ii Membrane Glycoprotein Related to the C-Type Lectin Superfamily. Eur J Immunol (1995) 25:2433–7. doi: 10.1002/eji.1830250904 7589107

[181] Hammer Q Ruckert T Borst EM Dunst J Haubner A Durek P. Peptide-Specific Recognition of Human Cytomegalovirus Strains Controls Adaptive Natural Killer Cells. Nat Immunol (2018) 19(5):453–63. doi: 10.1038/s41590-018-0082-6 29632329

[182] Lanier LL Corliss B Wu J Phillips JH. Association of Dap12 With Activating Cd94/Nkg2c Nk Cell Receptors. Immunity (1998) 8(6):693–701. doi: 10.1016/s1074-7613(00)80574-9 9655483

[183] Schlums H Cichocki F Tesi B Theorell J Beziat V Holmes TD. Cytomegalovirus Infection Drives Adaptive Epigenetic Diversification of Nk Cells With Altered Signaling and Effector Function. Immunity (2015) 42(3):443–56. doi: 10.1016/j.immuni.2015.02.008 PMC4612277 25786176

[184] Lee J Zhang T Hwang I Kim A Nitschke L Kim M. Epigenetic Modification and Antibody-Dependent Expansion of Memory-Like Nk Cells in Human Cytomegalovirus-Infected Individuals. Immunity (2015) 42(3):431–42. doi: 10.1016/j.immuni.2015.02.013 PMC4537797 25786175

[185] Hikami K Tsuchiya N Yabe T Tokunaga K. Variations of Human Killer Cell Lectin-Like Receptors: Common Occurrence of Nkg2-C Deletion in the General Population. Genes Immun (2003) 4(2):160–7. doi: 10.1038/sj.gene.6363940 12618865

[186] Miyashita R Tsuchiya N Hikami K Kuroki K Fukazawa T Bijl M. Molecular Genetic Analyses of Human Nkg2c (Klrc2) Gene Deletion. Int Immunol (2004) 16(1):163–8. doi: 10.1093/intimm/dxh013 14688071

[187] Liu LL Landskron J Ask EH Enqvist M Sohlberg E Traherne JA. Critical Role of Cd2 Co-Stimulation in Adaptive Natural Killer Cell Responses Revealed in Nkg2c-Deficient Humans. Cell Rep (2016) 15(5):1088–99. doi: 10.1016/j.celrep.2016.04.005 PMC4858565 27117418

[188] Moraru M Canizares M Muntasell A de Pablo R Lopez-Botet M Vilches C. Assessment of Copy-Number Variation in the Nkg2c Receptor Gene in a Single-Tube and Characterization of a Reference Cell Panel, Using Standard Polymerase Chain Reaction. Tissue Antigens (2012) 80(2):184–7. doi: 10.1111/j.1399-0039.2012.01911.x 22708664

[189] Goncalves A Makalo P Joof H Burr S Ramadhani A Massae P. Differential Frequency of Nkg2c/Klrc2 Deletion in Distinct African Populations and Susceptibility to Trachoma: A New Method for Imputation of Klrc2 Genotypes From Snp Genotyping Data. Hum Genet (2016) 135(8):939–51. doi: 10.1007/s00439-016-1694-2 PMC4947484 27312142

[190] Mellors JW Munoz A Giorgi JV Margolick JB Tassoni CJ Gupta P. Plasma Viral Load and Cd4+ Lymphocytes as Prognostic Markers of HIV-1 Infection. Ann Intern Med (1997) 126(12):946–54. doi: 10.7326/0003-4819-126-12-199706150-00003 9182471

[191] Mellors JW Rinaldo CR Jr. Gupta P White RM Todd JA Kingsley LA. Prognosis in HIV-1 Infection Predicted by the Quantity of Virus in Plasma. Science (1996) 272(5265):1167–70. doi: 10.1126/science.272.5265.1167 8638160

[192] Thomas R Low HZ Kniesch K Jacobs R Schmidt RE Witte T. Nkg2c Deletion Is a Risk Factor of HIV Infection. AIDS Res Hum Retroviruses (2012) 28(8):844–51. doi: 10.1089/AID.2011.0253 PMC3399562 22074011

[193] Gianella S Massanella M Wertheim JO Smith DM. The Sordid Affair Between Human Herpesvirus and HIV. J Infect Dis (2015) 212(6):845–52. doi: 10.1093/infdis/jiv148 PMC4548466 25748324

[194] Ma M Wang Z Chen X Tao A He L Fu S. Nkg2c(+)Nkg2a(-) Natural Killer Cells Are Associated With a Lower Viral Set Point and May Predict Disease Progression in Individuals With Primary HIV Infection. Front Immunol (2017) 8:1176. doi: 10.3389/fimmu.2017.01176 28979268 PMC5611385

[195] Rerks-Ngarm S Pitisuttithum P Nitayaphan S Kaewkungwal J Chiu J Paris R. Vaccination With Alvac and Aidsvax to Prevent HIV-1 Infection in Thailand. N Engl J Med (2009) 361(23):2209–20. doi: 10.1056/NEJMoa0908492 19843557

[196] Haynes BF Gilbert PB McElrath MJ Zolla-Pazner S Tomaras GD Alam SM. Immune-Correlates Analysis of an HIV-1 Vaccine Efficacy Trial. N Engl J Med (2012) 366(14):1275–86. doi: 10.1056/NEJMoa1113425 PMC3371689 22475592

[197] Gray GE Bekker LG Laher F Malahleha M Allen M Moodie Z. Vaccine Efficacy of Alvac-HIV and Bivalent Subtype C Gp120-Mf59 in Adults. N Engl J Med (2021) 384(12):1089–100. doi: 10.1056/NEJMoa2031499 PMC7888373 33761206

[198] Goodier MR Riley EM. Regulation of the Human Nk Cell Compartment by Pathogens and Vaccines. Clin Transl Immunol (2021) 10(1):e1244. doi: 10.1002/cti2.1244 PMC7813579 33505682

[199] Rydyznski CE Waggoner SN. Boosting Vaccine Efficacy the Natural (Killer) Way. Trends Immunol (2015) 36(9):536–46. doi: 10.1016/j.it.2015.07.004 PMC4567442 26272882

[200] Martin-Fontecha A Thomsen LL Brett S Gerard C Lipp M Lanzavecchia A. Induced Recruitment of Nk Cells to Lymph Nodes Provides Ifn-Gamma for T(H)1 Priming. Nat Immunol (2004) 5(12):1260–5. doi: 10.1038/ni1138 15531883

[201] Farsakoglu Y Palomino-Segura M Latino I Zanaga S Chatziandreou N Pizzagalli DU. Influenza Vaccination Induces Nk-Cell-Mediated Type-Ii Ifn Response That Regulates Humoral Immunity in an Il-6-Dependent Manner. Cell Rep (2019) 26(9):2307–15 e5. doi: 10.1016/j.celrep.2019.01.104 30811982

[202] Zwirner NW Domaica CI Fuertes MB. Regulatory Functions of Nk Cells During Infections and Cancer. J Leukoc Biol (2021) 109(1):185–94. doi: 10.1002/JLB.3MR0820-685R 33095941

[203] Cerwenka A Lanier LL. Natural Killer Cell Memory in Infection, Inflammation and Cancer. Nat Rev Immunol (2016) 16(2):112–23. doi: 10.1038/nri.2015.9 26806484

[204] Ochoa MC Minute L Rodriguez I Garasa S Perez-Ruiz E Inoges S. Antibody-Dependent Cell Cytotoxicity: Immunotherapy Strategies Enhancing Effector Nk Cells. Immunol Cell Biol (2017) 95(4):347–55. doi: 10.1038/icb.2017.6 28138156

[205] Li APY Cohen CA Leung NHL Fang VJ Gangappa S Sambhara S. Immunogenicity of Standard, High-Dose, Mf59-Adjuvanted, and Recombinant-Ha Seasonal Influenza Vaccination in Older Adults. NPJ Vaccines (2021) 6(1):25. doi: 10.1038/s41541-021-00289-5 33594050 PMC7886864

[206] Coccia M Collignon C Herve C Chalon A Welsby I Detienne S. Cellular and Molecular Synergy in As01-Adjuvanted Vaccines Results in an Early Ifngamma Response Promoting Vaccine Immunogenicity. NPJ Vaccines (2017) 2:25. doi: 10.1038/s41541-017-0027-3 29263880 PMC5627273

[207] Howard LM Hoek KL Goll JB Samir P Galassie A Allos TM. Cell-Based Systems Biology Analysis of Human As03-Adjuvanted H5n1 Avian Influenza Vaccine Responses: A Phase I Randomized Controlled Trial. PloS One (2017) 12(1):e0167488. doi: 10.1371/journal.pone.0167488 28099485 PMC5242433

[208] Cox A Cevik H Feldman HA Canaday LM Lakes N Waggoner SN. Targeting Natural Killer Cells to Enhance Vaccine Responses. Trends Pharmacol Sci (2021) 42(9):789–801. doi: 10.1016/j.tips.2021.06.004 34311992 PMC8364504

[209] Luetke-Eversloh M Hammer Q Durek P Nordstrom K Gasparoni G Pink M. Human Cytomegalovirus Drives Epigenetic Imprinting of the Ifng Locus in Nkg2chi Natural Killer Cells. PloS Pathog (2014) 10(10):e1004441. doi: 10.1371/journal.ppat.1004441 25329659 PMC4199780

[210] Zhang T Scott JM Hwang I Kim S. Cutting Edge: Antibody-Dependent Memory-Like Nk Cells Distinguished by Fcrgamma Deficiency. J Immunol (2013) 190(4):1402–6. doi: 10.4049/jimmunol.1203034 PMC3623944 23345329

[211] Rydyznski C Daniels KA Karmele EP Brooks TR Mahl SE Moran MT. Generation of Cellular Immune Memory and B-Cell Immunity Is Impaired by Natural Killer Cells. Nat Commun (2015) 6:6375. doi: 10.1038/ncomms7375 25721802 PMC4346304

[212] Rydyznski CE Cranert SA Zhou JQ Xu H Kleinstein SH Singh H. Affinity Maturation Is Impaired by Natural Killer Cell Suppression of Germinal Centers. Cell Rep (2018) 24(13):3367–73.e4. doi: 10.1016/j.celrep.2018.08.075 30257198 PMC6192537

[213] Moir S Fauci AS. B Cells in HIV Infection and Disease. Nat Rev Immunol (2009) 9(4):235–45. doi: 10.1038/nri2524 PMC2779527 19319142

[214] Buckner CM Kardava L Zhang X Gittens K Justement JS Kovacs C. Maintenance of HIV-Specific Memory B-Cell Responses in Elite Controllers Despite Low Viral Burdens. J Infect Dis (2016) 214(3):390–8. doi: 10.1093/infdis/jiw163 PMC4936645 27122593

[215] Bussmann BM Reiche S Bieniek B Krznaric I Ackermann F Jassoy C. Loss of HIV-Specific Memory B-Cells as a Potential Mechanism for the Dysfunction of the Humoral Immune Response Against HIV. Virology (2010) 397(1):7–13. doi: 10.1016/j.virol.2009.11.003 19962720

